# Forecasted trends in disability and life expectancy in England and Wales up to 2025: a modelling study

**DOI:** 10.1016/S2468-2667(17)30091-9

**Published:** 2017-05-23

**Authors:** Maria Guzman-Castillo, Sara Ahmadi-Abhari, Piotr Bandosz, Simon Capewell, Andrew Steptoe, Archana Singh-Manoux, Mika Kivimaki, Martin J Shipley, Eric J Brunner, Martin O'Flaherty

**Affiliations:** a Department of Public Health and Policy, University of Liverpool, Liverpool, UK; b Department Epidemiology and Public Health, University College London, London, UK; c Department of Prevention and Medical Education, Medical University of Gdansk, Gdansk, Poland; d Inserm U1018, Centre for Research in Epidemiology and Population Health, Villejuif, France

## Abstract

**Background:**

Reliable estimation of future trends in life expectancy and the burden of disability is crucial for ageing societies. Previous forecasts have not considered the potential impact of trends in disease incidence. The present prediction model combines population trends in cardiovascular disease, dementia, disability, and mortality to forecast trends in life expectancy and the burden of disability in England and Wales up to 2025.

**Methods:**

We developed and validated the IMPACT-Better Ageing Model—a probabilistic model that tracks the population aged 35–100 years through ten health states characterised by the presence or absence of cardiovascular disease, dementia, disability (difficulty with one or more activities of daily living) or death up to 2025, by use of evidence-based age-specific, sex-specific, and year-specific transition probabilities. As shown in the English Longitudinal Study of Ageing, we projected continuing declines in dementia incidence (2·7% per annum), cardiovascular incidence, and mortality. The model estimates disability prevalence and disabled and disability-free life expectancy by year.

**Findings:**

Between 2015 and 2025, the number of people aged 65 years and older will increase by 19·4% (95% uncertainty interval [UI] 17·7–20·9), from 10·4 million (10·37–10·41 million) to 12·4 million (12·23–12·57 million). The number living with disability will increase by 25·0% (95% UI 21·3–28·2), from 2·25 million (2·24–2·27 million) to 2·81 million (2·72–2·89 million). The age-standardised prevalence of disability among this population will remain constant, at 21·7% (95% UI 21·5–21·8) in 2015 and 21·6% (21·3–21·8) in 2025. Total life expectancy at age 65 years will increase by 1·7 years (95% UI 0·1–3·6), from 20·1 years (19·9–20·3) to 21·8 years (20·2–23·6). Disability-free life expectancy at age 65 years will increase by 1·0 years (95% UI 0·1–1·9), from 15·4 years (15·3–15·5) to 16·4 years (15·5–17·3). However, life expectancy with disability will increase more in relative terms, with an increase of roughly 15% from 2015 (4·7 years, 95% UI 4·6–4·8) to 2025 (5·4 years, 4·7–6·4).

**Interpretation:**

The number of older people with care needs will expand by 25% by 2025, mainly reflecting population ageing rather than an increase in prevalence of disability. Lifespans will increase further in the next decade, but a quarter of life expectancy at age 65 years will involve disability.

**Funding:**

British Heart Foundation.

## Introduction

The substantial expansion in life expectancy and population ageing during the 20th century is continuing into the 21st century. Life expectancy at age 65 years among the 27 countries of the European Union has increased from 17·8 years in 2002, to 20·0 years in 2014.^1^ Rapid ageing of populations in developed countries is set to continue; however, evidence about trends in morbidity and disability prevalence in the past few decades is inconsistent.^2^, ^3^

Policy makers, service planners, and clinicians need reliable forecasts of future trends in life expectancy and the burden of disease and disability. Current projections involve simple extrapolations that fail to consider the combined effect that trends in disease incidence, particularly cardiovascular disease and dementia, will have on the health status of older people. In the UK, concerns exist regarding potential increases in age-related disability. Between 1991 and 2011, findings from the Cognitive Function and Ageing Study (CFAS)^4^ showed that although total life expectancy and disability-free life expectancy increased, the proportion of life without disability decreased.

Trends in life expectancy and disability are shaped primarily by trends in the burden of cardiovascular disease and dementia.^5^, ^6^, ^7^ Both conditions are important underlying causes of age-related disability, particularly in middle-income and high-income countries.^7^ Cardiovascular disease morbidity and mortality have fallen greatly in the past few decades.^8^, ^9^ The associated prolongation of life expectancy has enlarged the pool of individuals surviving to old age and hence susceptible to dementia. Furthermore, because dementia and cardiovascular disease share behavioural and biomedical risk factors,^5^, ^10^ reduction in vascular risk might also reduce age-specific dementia incidence.^11^, ^12^, ^13^ On the basis of these two opposing effects, forecasting of the projected prevalence of disability requires simultaneous modelling of both conditions.

**Research in context**

**Evidence before this study**

Between Oct 1, and Oct 30, 2016, we searched PubMed for studies forecasting future trends in disability or dementia in the UK, with the search terms “disability”, “dementia”, “longevity”, “life expectancy”, “forecasting”, “simulation”, “model” and synonyms of “United Kingdom”. The appendix (pp 12, 13) lists our complete PubMed search strings and shows results of our systematic review of the literature. We did additional hand searches with lists of references retrieved from relevant papers. We identified only two studies forecasting total life expectancy at age 65 years, neither of which investigated disability or disability-free life expectancies, and two studies reporting a future number of cases with disability in England and Wales. None of these studies modelled future trends in disability and life expectancy while explicitly including interactions between trends in cardiovascular disease and dementia.

**Added value of this study**

To our knowledge, this is the first study to model future trends in disability in the UK using empirical longitudinal data for England and Wales while also taking into account interactions over time between cardiovascular disease, dementia, and disability. Our findings show that people in England and Wales will live longer but, on average, a quarter of the extra years gained after age 65 years will involve disability. The overall burden of disability will grow primarily as a consequence of population ageing rather than an increase in the prevalence of disability. These predictions have profound individual and societal implications.

**Implications of all available evidence**

Changes in vascular risk factors are considered to be the primary drivers of trends in cardiovascular disease and dementia incidence; therefore, future forecasts of disability need to take into account the interaction of these conditions over time. Simulation modelling offers a platform to gain new insights to inform these projections and highlight opportunities for further refinement. Subsequent research should identify which prevention strategies might provide the biggest health and economic benefits in the future.

Previous studies have not considered the complex synergies of life expectancy, cardiovascular disease, and dementia, nor the contribution of these chronic conditions to disability over time.^14^ We therefore aimed to forecast trends in disability and life expectancy in England and Wales up to 2025, simultaneously accounting for time-varying trends in morbidity and mortality.

## Methods

### Model design

We developed and validated the IMPACT-Better Ageing Model (IMPACT-BAM)—a discrete-time probabilistic Markov model that follows the progression of a healthy population (aged 35–100 years) of England and Wales from 2006 to 2025 into eight different states characterised by the presence or absence of cardiovascular disease, cognitive impairment, and moderate-to-severe functional impairment (moderate-to-severe disability), and two states for death from cardiovascular disease and non-cardiovascular disease causes (appendix p 14). Movements of the population between these ten states are governed by 1 year age-specific, sex-specific, and year-specific probabilities of transition. IMPACT-BAM is a population model such that for each year in the simulation, a new cohort of 35-year-olds enters the system through the disease-free state.

### Data sources

We combined age-specific and sex-specific population estimates from the Office for National Statistics with prevalence data from the English Longitudinal Study of Ageing (ELSA)^15^ to populate all the states in the model at the start of the simulation. We used projections from the Office for National Statistics until 2025 to create the input population vector of 35-year-olds assumed to be disease-free at entry. Data for calculation of probabilities of transition were also from ELSA.^15^

### Health states

We defined cardiovascular disease as a diagnosis of cardiovascular disease; myocardial infarction; or stroke or angina, or both. We defined cognitive impairment without dementia as impairment in two or more domains of cognitive function tests applied to ELSA participants (such as orientation to time, immediate and delayed memory, verbal fluency, and numeracy function). For individuals who were unable to take the cognitive function tests, the Informant Questionnaire on Cognitive Decline (IQCODE) was administered to a proxy informant (usually an immediate family member).^16^ A score higher than 3·6 on the IQCODE was used to identify cognitive impairment without dementia. We defined functional impairment or disability as the inability to independently do one or more activities of daily living, which included getting in or out of bed, walking across a room, bathing or showering, using the toilet, dressing, cutting food, and eating. This definition of disability captures numbers of individuals who have difficulty maintaining self-care independence and require supportive care.

We defined dementia on the basis of the coexistence of cognitive impairment and functional impairment, or a report of a doctor diagnosis of dementia by the participant or caregiver. Transitions to the two death states (cardiovascular disease or non-cardiovascular disease causes) were possible from any health state (appendix p 14). We distinguished four disability states: cardiovacular disease-related disability; dementia-related disability; cardiovacular disease-related and dementia-related disability; and other disease-related disability, defined as other forms of disability not linked to cardiovascular disease or dementia.

### Model assumptions and outputs

We assumed that population trends in cardiovascular disease incidence would parallel the rate of decline in cardiovascular disease mortality up to 2025, as observed in ELSA for the period 2002–13.^15^ We further assumed that dementia incidence would follow a 2·7% relative annual decline, as also observed in ELSA.

IMPACT-BAM was used to calculate future trends in the prevalence of disability (both age-specific and age-standardised using the population in 2015), life expectancy, disabled life expectancy, and disability-free life expectancy according to the Sullivan method.^17^ The model was implemented as a statistical package in R software. Appendix pp 13–23 provide detailed information about data sources, definitions of health states, and methods.

### Sensitivity analyses

In view of uncertainty regarding trends in dementia incidence, we tested two alternative assumptions for the trend in future dementia incidence: constant incidence (no annual decline) and an annual decline of 4% in dementia incidence (appendix pp 24, 25).

To explore the effect of parameter uncertainty on model outputs, we did a probabilistic sensitivity analysis using Monte Carlo simulation. The procedure entailed iterative sampling from specified distributions for the input parameters used in the model and recalculation of the outputs. We did 1000 iterations to estimate 95% uncertainty intervals (UIs) for the output variables. Appendix p 25 provides additional details of distributions and statistical functions.

### Model validation

To validate cardiovascular disease and non-cardiovascular disease deaths, we compared our model estimates with observed mortality data from the Office of National Statistics for the period 2006–12. For cardiovascular disease prevalence, we compared our model estimates with those published by the Health Survey for England 2011. For disability prevalence, we compared 2014 prevalence estimates from the most recent wave in ELSA (not used in the design of the model) with our estimates. For dementia prevalence, we compared our model estimates with those reported in the CFAS II.^3^ Finally, we compared our estimates of life expectancy at age 65 years for the period 2006–12 with those reported by the European Health and Life Expectancy Information System (EHLEIS) and the Office of National Statistics (appendix pp 27–31).

### Role of the funding source

The funder of the study had no role in study design, data collection, data analysis, data interpretation, or writing of the report. The corresponding author had full access to all the data in the study and final responsibility for the decision to submit for publication.

## Results

Our projections indicate that the number of people in England and Wales aged 65 years and older will increase by roughly 19·4% (95% UI 17·7–20·9), from 10·4 million (10·37–10·41 million) in 2015, to 12·4 million (12·23–12·57 million) in 2025. Notably, the number of people aged 85 years and older will increase by 38·9% (95% UI 31·9–45·5), from 1·4 million (1·38–1·45 million) in 2015, to 2·0 million (1·87–2·07 million) in 2025.

**Figure 1:**
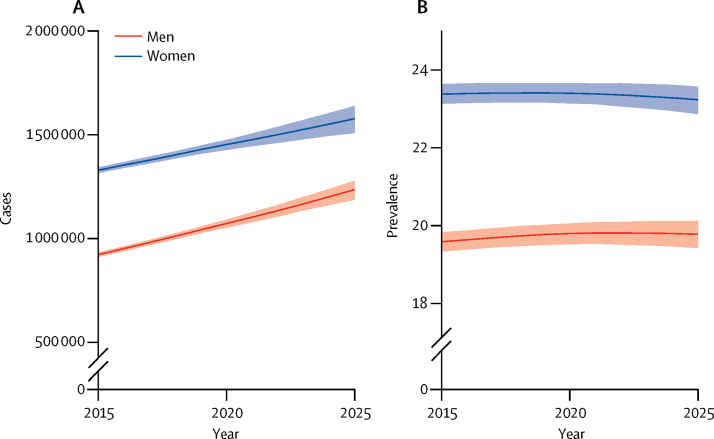
Projected number of cases (A) and prevalence (B) of disability in men and women aged 65 years or older from 2015 to 2025 in England and Wales Shaded areas represent 95% uncertainty intervals. Prevalence of disability is standardised to 2015 population estimates for England and Wales.

The age-specific disability prevalence between 2002 and 2013 in ELSA ranged from 14% in people aged 65–69 years to 57% in those aged 90 years and older (appendix p 26). Additionally, we estimated that 53% of all people aged 65 years or older with disability had cardiovascular disease or cognitive impairment (appendix pp 26, 27). We predict that, between 2015 and 2025, the number of people living with disability will increase by about 2·3% per annum (table 1). By 2025, roughly 2·8 million people will be living with disability, an additional 560 000 cases compared with 2015 (a 25·0% overall increase; table 1). The number of men living with disabilities will increase by roughly 3% per year to reach 1·24 million cases in 2025, whereas the number of women living with disabilities will increase by 1·7% per year to reach 1·58 million cases in 2025 (table 1, figure 1).

**Table 1:** Projected number of disability cases (in thousands) by sex and age in 2015 and 2025 in England and Wales

	**2015**	**2025**	**Annual relative change (%)**	**Relative change between 2015 and 2025 (%)**
**All people (age, years)**				
≥65	2251 (2235–2268)	2811 (2727–2890)	2·3% (2·0–2·5)	25·0% (21·3–28·2)
65–84	1692 (1679–1706)	2010 (1969–2049)	1·7% (1·5–1·9)	18·9% (16·6–20·9)
≥85	559 (552–567)	800 (750–852)	3·7% (3·0–4·3)	43·2% (34·2–52·1)
**Men (age, years)**				
≥65	922 (911–933)	1236 (1187–1279)	3·0% (2·5–3·3)	34·0% (28·6–38·5)
65–84	745 (735–755)	914 (888–939)	2·1% (1·8–2·3)	22·6% (19·2–25·5)
≥85	177 (173–181)	322 (297–349)	6·1% (5·3–6·9)	81·6% (67·2–95·3)
**Women (age, years)**				
≥65	1329 (1316–1343)	1578 (1508–1639)	1·7% (1·3–2·1)	18·7% (13·7–23·2)
65–84	947 (936–959)	1098 (1068–1126)	1·5% (1·2–1·7)	16·0% (12·9–18·8)
≥85	382 (376–388)	480 (434–521)	2·3% (1·3–3·2)	25·4% (14·2–36·4)

Data in parentheses are 95% uncertainty intervals.

Between 2015 and 2025, other disease-related disability will remain the most frequent type of disability among people aged 65–84 years, whereas dementia-related and cardiovascular disease-related disability will persist as the most common types of disability among people aged 85 years or older (table 2). The numbers of other disease-related disability cases among people aged 65–84 years will increase in the next decade by about 31%, and the number of dementia-related disability cases by about 40%; however, the cases of cardiovascular disease-related disability will decline by about 17% (table 2). In people aged 85 years or older, the numbers of other disease-related disability, dementia-related disability, and cardiovascular disease-related disability will increase by 84%, 63%, and 6%, respectively (table 2).

**Table 2:** Projected number of disease-related disability cases (in thousands) by age in 2015 and 2025 in England and Wales

	**2015**	**2025**	**Relative change between 2015 and 2025 (%)**
**≥65 years old**			
CVD-related disability	588 (576 to 599)	527 (505 to 547)	−10·3% (−13·6 to 7·3)
Dementia-related disability	468 (447 to 491)	699 (654 to 745)	49·1% (41·5 to 56·2)
Dementia and CVD-related disability	177 (171 to 183)	191 (178 to 203)	7·7% (1·3 to 14·0)
Other disease-related disability	1018 (995 to 1041)	1395 (1355 to 1440)	37·1% (34·3 to 39·8)
**65 to 84 years old**			
CVD-related disability	419 (410 to 428)	348 (335 to 363)	−16·9% (−19·8 to 3·9)
Dementia-related disability	289 (269 to 309)	405 (374 to 436)	40·3% (34·7 to 46·3)
Dementia and CVD-related disability	84 (79 to 88)	79 (73 to 85)	−5·3% (−10·8 to 0·7)
Other disease-related disability	900 (879 to 921)	1177 (1145 to 1213)	30·9% (28·5 to 33·1)
**≥85 years old**			
CVD -related disability	168 (163 to 174)	179 (166 to 190)	6·0% (−0·8 to 12·6)
Dementia-related disability	179 (171 to 188)	293 (267 to 320)	63·1% (50·7 to 76·5)
Dementia and CVD-related disability	93 (89 to 97)	111 (102 to 120)	19·3% (9·8 to 29·0)
Other disease-related disability	118 (111 to 126)	217 (200 to 235)	84·2% (74·6 to 94·2)

Data in parentheses are 95% uncertainty intervals. CVD=cardiovascular disease.

The age-standardised prevalence of disability in the population aged 65 years or older will remain broadly constant to 2025 in both men and women (figure 1). A modest increase of 1·6% in prevalence is predicted in the oldest men in the next decade (table 3); however, trends in age-standardised prevalence will differ by type of disability: prevalence of cardiovascular disease-related disability will decline by about 30%, whereas prevalence of dementia-related and other disease-related disability will increase by about 14% (appendix pp 4–6).

**Table 3:** Projected percentage of disability cases by sex and age in 2015 and 2025 in England and Wales

	**2015**	**2025**
**All people (age, years)**		
≥65	21·7% (21·5–21·8)	21·6% (21·3–21·8)
≥75	28·8% (28·5–29·0)	29·0% (28·5–29·3)
≥85	39·5% (38·9–40·0)	39·8% (39·0–40·4)
**Men (age, years)**		
≥65	19·6% (19·3–19·8)	19·8% (19·4–20·1)
≥75	25·6% (25·2–26·0)	26·4% (25·9–26·9)
≥85	34·8% (34·0–35·7)	36·4% (35·4–37·3)
**Women (age, years)**		
≥65	23·4% (23·1–23·6)	23·2% (22·9–23·6)
≥75	31·1% (30·7–31·4)	31·0% (30·4–31·5)
≥85	42·1% (41·4–42·8)	42·2% (41·2–43·0)

Data in parentheses are 95% uncertainty intervals. Percentages are age-standardised to the 2015 population of England and Wales.

**Figure 2:**
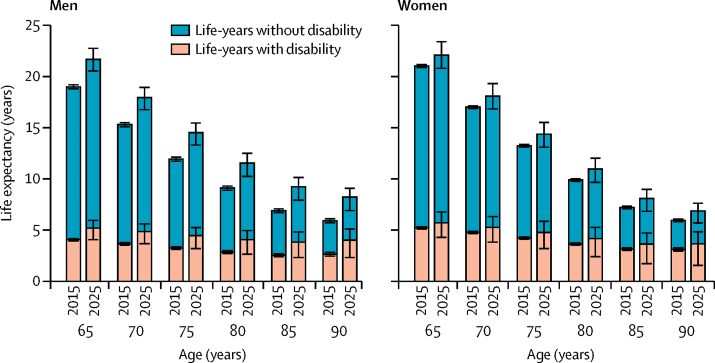
Projected life expectancy trends between 2015 and 2025 in England and Wales decomposed by years lived with and without disability from different age points Error bars show 95% uncertainty intervals.

Overall in people aged 65 years, total life expectancy, disability-free life expectancy, and life expectancy with disability will increase in the entire population between 2015 and 2025 (table 4), and there will be increases in life expectancy in all age groups (figure 2). Appendix p 31 compares the life expectancy estimates in our study with those from other studies. Disability-free life expectancy at age 65 years will increase by 1·0 years, from 15·4 years to 16·4 years; however, life expectancy with disability will grow more in relative terms (about 15% increase from 2015; table 4). For both health expectancies, bigger increases are predicted for men than for women (table 4). Overall, the proportion of the life expectancy lived with disabilities at age 65 years will increase from 21·4% to 24·0% in men and 24·9% to 25·8% in women (table 4).

**Table 4:** Projected estimates of life expectancy in people aged 65 years in 2015 and 2025 in England and Wales

	**2015**	**2025**	**Difference**
**All people**			
Total	20·1 (19·9 to 20·3)	21·8 (20·2 to 23·6)	1·7 (0·1 to 3·6)
Without disability	15·4 (15·3 to 15·5)	16·4 (15·5 to 17·3)	1·0 (0·1 to 1·9)
With disability	4·7 (4·6 to 4·8)	5·4 (4·7 to 6·4)	0·7 (0·0 to 1·7)
Proportion lived with disability	23·4% (23·1 to 23·6)	24·9% (23·2 to 27·0)	1·5% (−0·1 to 3·7)
**Men**			
Total	19·0 (18·7 to 19·3)	21·7 (19·9 to 23·9)	2·7 (0·9 to 4·9)
Without disability	14·9 (14·7 to 15·1)	16·5 (15·4 to 17·6)	1·6 (0·5 to 2·7)
With disability	4·1 (3·9 to 4·2)	5·2 (4·4 to 6·3)	1·1 (0·4 to 2·2)
Proportion lived with disability	21·4% (21·0 to 21·7)	24·0% (22·2 to 26·4)	2·6% (0·8 to 5·0)
**Women**			
Total	21·0 (20·8 to 21·2)	22·1 (19·7 to 24·7)	1·1 (−1·3 to 3·8)
Without disability	15·8 (15·7 to 15·9)	16·4 (15·1 to 17·7)	0·6 (−0·7 to 1·9)
With disability	5·2 (5·1 to 5·3)	5·7 (4·6 to 7·1)	0·5 (−0·6 to 1·9)
Proportion lived with disability	24·9% (24·5 to 25·2)	25·8% (23·5 to 28·9)	0·9% (−1·4 to 4·1)

Data in parentheses are 95% uncertainty intervals.

Results of our sensitivity analysis showed that estimates of numbers of people with disability remained almost unchanged despite different calendar trends in incidence of dementia (appendix p 8). Furthermore, the proportion of life expectancy lived with disability will remain virtually unchanged from the baseline scenario for both men and woman (appendix p 11). However, the two alternative assumptions regarding the trend in future dementia incidence do affect the numbers in the disease-specific disability (appendix pp 7–11). If dementia incidence remains unchanged over the next decade, the cases of dementia-related disability will increase compared to our main prediction. This increase will be counterbalanced by a decrease in the number of cases of other types of disability, including cardiovascular disease-related disability. Conversely, a faster annual decline in dementia incidence would result in fewer cases of dementia-related disability, but an increase in the numbers of other types of disability.

We validated key model outputs against empirical observations. The model provided a good match with the Office for National Statistics estimates of the number of cardiovascular and non-cardiovascular deaths (appendix pp 28, 29). Our estimates of cardiovascular disease prevalence in 2011 were similar to those reported by the Health Survey England, particularly for men (appendix p 29). Our findings for disability prevalence in 2014 for women were very similar to those reported by ELSA wave 7; however, our model estimates a slightly lower prevalence of disability in men (appendix p 30). Our age-specific estimates of dementia in 2011 were similar to those reported in CFAS II (appendix p 30). Our estimates of life expectancy at 65 years for 2006–15 were similar to those reported by the Office of National Statistics and EHLEIS (appendix pp 30, 31).

## Discussion

Our life expectancy and disability forecasts are based on a Markov model, which, for the first time, synthesises the combined effects of present trends in incidence of cardiovascular disease, dementia disability, and mortality. Our findings show that the number of people aged 65 years or older with care needs in England and Wales could reach 2·8 million by 2025. This 25% increase will mainly reflect population ageing rather than an increase in the prevalence of disability. Lifespans will increase further, but a quarter of life expectancy at age 65 years will involve disability.

Other forms of disability not linked to cardiovascular disease or dementia will persist between 2015 and 2025 as the most frequent type of disability among people aged 65–84 years. Our findings are consistent with those from a 2016 analysis of the most important contributors to global disability burden for this age group.^18^ For people older than 85 years, future disability levels will be influenced mainly by the joint evolution of the burdens of dementia and cardiovascular disease over time. Evidence suggests that declines in cardiovascular disease incidence and mortality in the past few decades are set to continue.^8^, ^9^ Encouragingly, progressive declines in the incidence of dementia have been reported in the past 20 years in Europe and the USA;^11^, ^12^, ^13^ however, the size of the decline varied across these study populations. To account for this uncertainty, we modelled the 2·7% annual decline observed in ELSA^15^ and showed that the projected burden of disability was robust to two alternative assumptions about the future dementia incidence trend, 0% and 4%.

Our projections of life expectancy at 65 years are broadly similar to those from the Office of National Statistics and other studies;^19^, ^20^ the largest differences are for women, for whom our projections are lower. In developed countries, women have tended to live longer, but to have worse health than men.^21^ Excess mortality in men has been attributed to higher prevalence of life-threatening chronic conditions such as cardiovascular disease.^22^ Therefore, the higher increment in life expectancy for men than for women between 2015 and 2025, could be explained by life expectancy in men being more sensitive to the declining trends in cardiovascular disease incidence and mortality observed in ELSA over the past decade and projected forward in our model. Although women have poorer health than men, attributable to a higher prevalence of dementia and functional limitations, our results suggest the present sex difference in disabled life expectancy will diminish because men will have a relatively larger increase in the burden of disability.

Two previous studies have predicted future numbers of disability cases in England and Wales. Jagger and colleagues^23^ used a macro-simulation model of cohort transitions between non-disabled, disabled, and death states from 1992 to 2026. The investigators estimated transition probabilities from CFAS I conditional on the prevalence of several chronic conditions and assuming their prevalence to be constant over the forecasting period. Comas-Herrera and colleagues^24^ applied age-specific and sex-specific prevalence of cognitive impairment and disability observed in 2002 (also based on CFAS I) to Government Actuary Department's population projections in 2031, again assuming that dementia incidence will be constant over time. Comparisons of these studies with ours are indirect because the definitions of disability differ. Correspondingly, both previous studies predicted a smaller future burden of disability than IMPACT-BAM. This divergence arises largely because estimates of disability prevalence in the base year of respective models (CFAS I: 1992, ELSA: 2002) depend on the number of activities included in the activities of daily living instrument. The ELSA instrument included six activities, compared with three in CFAS I.^25^ The greater the number of items of activities of daily living, the higher the probability of detection of disabilities.

Credible predictions of future prevalence of disability and life expectancy need to consider the complex population dynamics of lifespan and the major disability determinants. IMPACT-BAM responds to these requirements by modelling two major diseases with shared and strongly interrelated determinants, both of which are associated with substantial functional impairment.^5^, ^10^ Few studies have attempted to estimate future disability in England and Wales on the basis of dementia trends.^23^, ^24^ Moreover, none has explicitly considered transitions between cardiovascular disease, dementia, and functional impairment states while simultaneously taking into account changing time trends in dementia incidence, cardiovascular disease incidence, cardiovascular disease mortality, and non-cardiovascular disease mortality. Furthermore, our model parameters derive from the best available population-based longitudinal evidence in the UK, and cross-validation of our projections used external data sources and estimates.

Prediction modelling has limitations. First, IMPACT-BAM aggregates disability caused by conditions such as musculoskeletal disorders, mental disorders, diabetes, chronic respiratory diseases, and other chronic diseases in a single other disease-related disability state. Transitions from this state to other states in the model are not conditioned to the prevalence of these specific chronic conditions. However, because ELSA is representative of the English population, the estimated transition probabilities to cardiovascular disease, dementia, or death from this combined state are likely to represent a weighted average of risk across all comorbidities. Although outside the scope of our study, consideration of disability on a spectrum of severity has important implications for health-care planning and policy, because each level of severity generates corresponding needs in care and assistance. Our binary definition of disability is relevant to future demand of long-term care because it provides a transparent basis for estimation of the trend in numbers of individuals who would require supportive care. Finally, the cohort of 35-year-olds populating the model every year was obtained from official population projections.^26^ These principal projections are based on assumptions regarding future levels of fertility, migration, and mortality, which might add uncertainty to our estimates. However, population projections have proved to be relatively robust to mortality assumptions, whereas fertility and migrant variant assumptions only affect the projected numbers of children and young adults.^26^

The societal, economic, and public health implications of our forecast are substantial. In particular, our findings draw attention to the scale of societal costs associated with disability in the coming decade. Public and private expenditure on long-term care will need to increase considerably by 2025, in view of the predicted 25% rise in the number of people who will have age-related disability. This situation has serious implications for a cash-strapped and overburdened National Health Service, and an under-resourced social care system.

In the context of the rapid and continuing rise in the number of older dependent people in the UK, the government needs to give urgent consideration to options for more cost-effective health and social care provision in all its forms. First, national capacity for institutional care needs to increase. Second, informal and home care require stronger policy support, for example by means of tax allowances or cash benefits. Affected individuals and their families pay an estimated 40% of the national cost of long-term care from income and savings.^27^ Notably, the disadvantage of older people on lower incomes unable to live independently will increase if the shortage of caregivers and the precarious state of institutional and domiciliary care provision is not addressed.^28^

Cardiovascular disease and dementia share risk factors—namely, poor diet, smoking, high alcohol consumption, hypertension, diabetes, and physical inactivity. Effective prevention strategies are therefore strongly advocated by UK health charities, the National Institute of Health and Care Excellence, and WHO.^29^, ^30^ This shared-determinants approach is an important but neglected strategy for reducing the future burden of disability at the population level. Immediate substantial investment in such prevention policies could be substantially cost-saving.^31^ Policy simulation modelling based on interventions aimed at the drivers of disability is needed to inform this debate. Potential prevention strategies include healthy food interventions, fiscal measures for obesity control, and disease-specific health-care-based interventions.

In conclusion, our evidence-based forecasting model, incorporating the expected continuing declines in cardiovascular disease and dementia incidence, predicts that the number of people living with disability will increase over the next decade. This increase mainly reflects the expected continuing rise in life expectancy and resulting upward shift in the age distribution of the population. The rising burden of age-related disability accompanying population ageing poses a substantial societal challenge and emphasises the urgent need for policy development that includes effective prevention interventions.
