# Supplementary material for: Forecasted trends in disability and life expectancy in England and Wales up to 2025: a modelling study
Source: Lancet Public Health. 2017 May 23;2(7):e307–13. doi: 10.1016/S2468-2667(17)30091-9 (PMC5500313; doi:10.1016/S2468-2667(17)30091-9)
Supplement: Supplementary appendix [file mmc1.pdf]

THE LANCET
Public Health

Supplementary appendix

This appendix formed part of the original submission and has been peer reviewed. We post it as supplied by the authors.

Supplement to: Guzman-Castillo M, Ahmadi-Abhari S, Bandosz P, et al. Forecasted trends in disability and life expectancy in England and Wales up to 2025: a modelling study. *Lancet Public Health* 2017; published online May 23. http://dx.doi.org/10.1016/S2468-2667(17)30091-9.

Supplementary Material

Forecasting trends in disability and life expectancies in England and Wales to 2025: a modelling study

Maria Guzman Castillo[1] PhD, Sara Ahmadi-Abhari[2] PhD, Piotr Bandosz[1,3] PhD, Professor Simon Capewell [1] DSc, Professor Andrew Steptoe[2] DSc, Professor Archana Singh-Manoux[2,4] PhD , Professor Mika Kivimaki[2] PhD, Martin J Shipley[2] MSc, Professor Eric J Brunner[2], PhD* Professor Martin O'Flaherty[1] PhD*

*Joint senior authors

1. Department of Public Health and Policy, University of Liverpool, Liverpool, United Kingdom;
2. Department Epidemiology & Public Health, University College London, UK
3. Department of Prevention and Medical Education, Medical University of Gdansk, Gdansk, Poland
4. Inserm, U1018, Centre for Research in Epidemiology and Population Health, Villejuif, France

Correspondence:

mdlgc106@liverpool.ac.uk

Keywords: life expectancy, disability, dementia, cardiovascular, forecast.

Funding

British Heart Foundation (RG/13/2/30098)

Contents

Supplementary Material 1

Forecasting trends in disability and life expectancies in England and Wales to 2025: a modelling study 1

1 Detailed Results 4

2 Sensitivity analysis 7

3 Research in context 12

4 Overview of IMPACT Better Aging Model 13

5 Inputs and Calculations 16

5.1 Case definitions 16

5.2 Incidence of CVD ($P_{1,2}$; $P_{4,3,}$ $P_{8,5}$) 17

5.3 Incidence of CIND ($P_{1,4}$; $P_{2,3,}$ $P_{8,7}$) 17

5.4 Incidence of CVD and CIND ($P_{1,3}$; $P_{8,6}$) 18

5.5 Incidence of functional impairment states ($P_{1,8}$; $P_{2,5;}$ $P_{3,6 \text{ and }}$ $P_{4,7}$) 18

5.6 Recovery from functional impairment states ($P_{8,1}$; $P_{5,2}$; $P_{6,3}$ and $P_{7,4}$) 18

5.7 Transition probabilities from state i to the death states ($P_{i,9 \text{ and }}$ $P_{i,10}$) 18

5.8 Calendar effect for CVD and CIND incidence 19

5.9 Recurrent state transition probabilities 20

5.10 Prevalence of initial states 21

6 Matrix calculations 21

7 Output statistics from IMPACT-BAM 22

8 Probability sensitivity analysis: Monte Carlo simulation 24

8.1 Basic Monte Carlo simulation 24

9 Prevalence of disability from ELSA 26

10 Validation of the model 27

10.1 CVD mortality 28

10.2 Non-CVD mortality 29

10.3 Prevalence of CVD 29

10.4 Prevalence of disability 30

10.5 Prevalence of dementia 30

10.6 Life expectancy 30

11 References 32

1 Detailed Results

Table S. 1: Predicted cases of disability 2015-2025. (95% uncertainty intervals)

Year	All persons	Men	Women
**2015**	2,251,225 (2,234,794-2,267,986)	922,198 (911,016-932,855)	1,329,147 (1,316,237-1,342,710)
**2016**	2,305,968 (2,288,503-2,323,692)	951,815 (939,714-963,034)	1,354,398 (1,340,333-1,368,847)
**2017**	2,358,618 (2,340,071-2,379,518)	981,131 (968,316-993,900)	1,377,602 (1,362,379-1,394,832)
**2018**	2,413,948 (2,392,030-2,435,340)	1,011,248 (996,556-1,025,173)	1,402,890 (1,385,718-1,419,979)
**2019**	2,470,933 (2,441,840-2,495,505)	1,042,121 (1,025,662-1,058,276)	1,428,961 (1,408,509-1,448,636)
**2020**	2,526,136 (2,492,134-2,555,829)	1,072,611 (1,053,071-1,091,239)	1,453,139 (1,427,172-1,475,253)
**2021**	2,579,557 (2,542,306-2,617,358)	1,103,144 (1,078,727-1,126,678)	1,476,335 (1,445,324-1,506,401)
**2022**	2,633,744 (2,586,828-2,680,767)	1,134,485 (1,105,974-1,161,545)	1,500,500 (1,460,971-1,537,922)
**2023**	2,693,231 (2,636,494-2,750,364)	1,167,276 (1,135,941-1,200,504)	1,525,727 (1,478,254-1,571,590)
**2024**	2,753,674 (2,679,587-2,818,366)	1,201,302 (1,160,976-1,238,509)	1,551,329 (1,495,101-1,604,529)
**2025**	2,811,053 (2,727,384-2,889,965)	1,236,268 (1,187,308-1,278,668)	1,577,604 (1,508,284-1,638,884)

Table S. 2: Predicted crude prevalence of disability 2015-2025 (95% uncertainty intervals)

Year	All persons	Men	Women
**2015**	21.7 (21.5 - 21.8)	19.6 (19.3 - 19.8)	23.4 (23.1 - 23.6)
**2016**	21.8 (21.6 – 22.0)	19.8 (19.5 20.0)	23.5 (23.2 - 23.7)
**2017**	21.9 (21.7 - 22.1)	20.0 (19.7 - 20.2)	23.6 (23.3 - 23.8)
**2018**	22.0 (21.9 - 22.2)	20.1 (19.9 - 20.4)	23.6 (23.4 - 23.9)
**2019**	22.2 (22.0 - 22.4)	20.3 (20.1 - 20.6)	23.7 (23.5 -24.0)
**2020**	22.3 (22.1 - 22.5)	20.5 (20.2 - 20.8)	23.8 (23.5 - 24.1)
**2021**	22.4 (22.2 - 22.6)	20.7 (20.4- 21.0)	23.9 (23.6 - 24.2)
**2022**	22.5 (22.2 - 22.8)	20.8 (20.5 - 21.2)	23.9 (23.5 - 24.3)
**2023**	22.6 (22.3 - 22.9)	21.0 (20.6 - 21.3)	24.0 (23.5 - 24.4)
**2024**	22.6 (22.3 - 22.9)	21.1 (20.7 - 21.5)	24.0 (23.5 - 24.5)

**2025**	22.7 (22.3 - 23)	21.2 (20.7 - 21.6)	24.0 (23.4 - 24.5)

Our model predicts that the age-standardised prevalence of disability in the population aged over 65 will remain broadly constant to 2025 in both men and women. However, differing trends are revealed when looking at disease-related disability states. The age-standardised prevalence of CVD-related disability will decrease in men and women between 2015 and 2025, following the declines in CVD incidence and mortality (see red line in panel B of Figure S. 1 and Figure S. 2, included below). In contrast, the age-standardised prevalence of dementia-related disability and other disease-related disability will both increase between 2015 and 2025.

Figure S. 1: Projected number of cases (A) and age-standardised prevalence (B) of disease-related disability in men aged≥65 years from 2015 to 2025 in England and Wales.

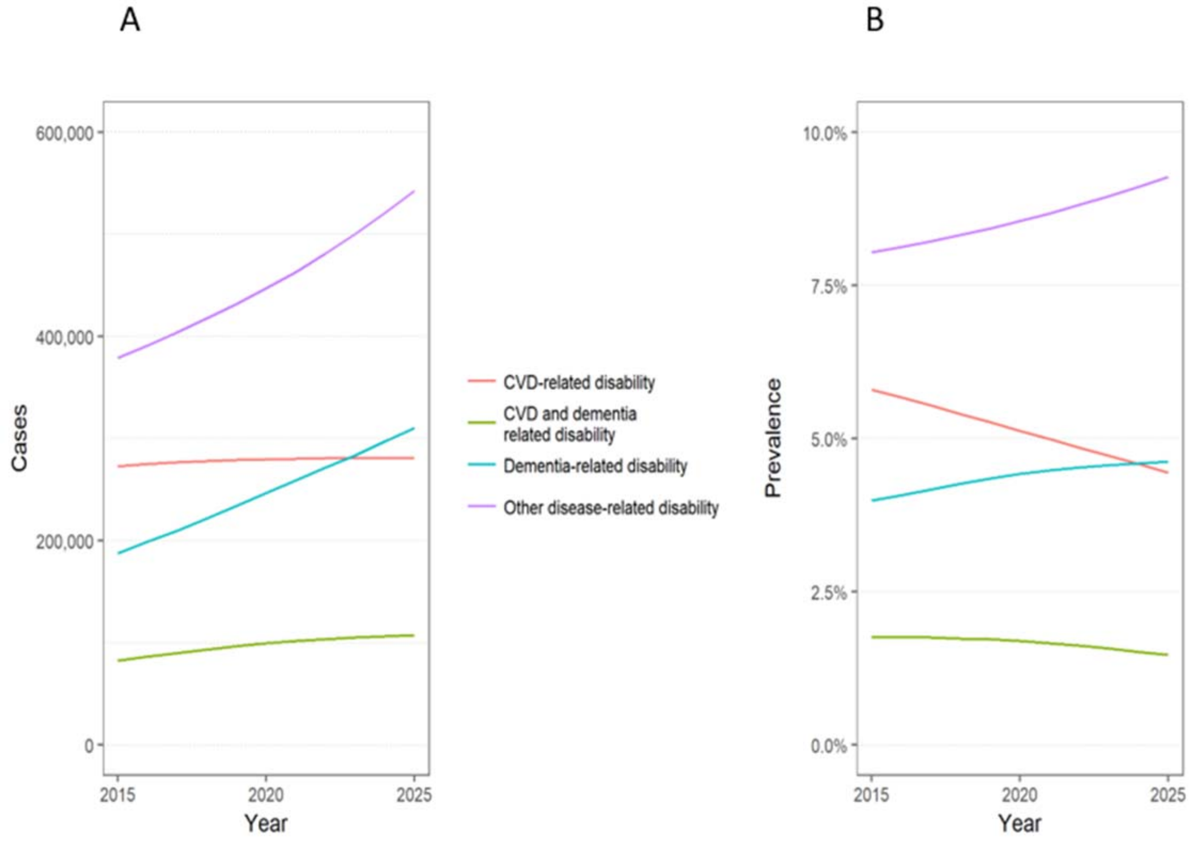

Figure S. 2: Projected number of cases (A) and age-standardised prevalence (B) of disease-related disability in women aged≥65 years from 2015 to 2025 in England and Wales.

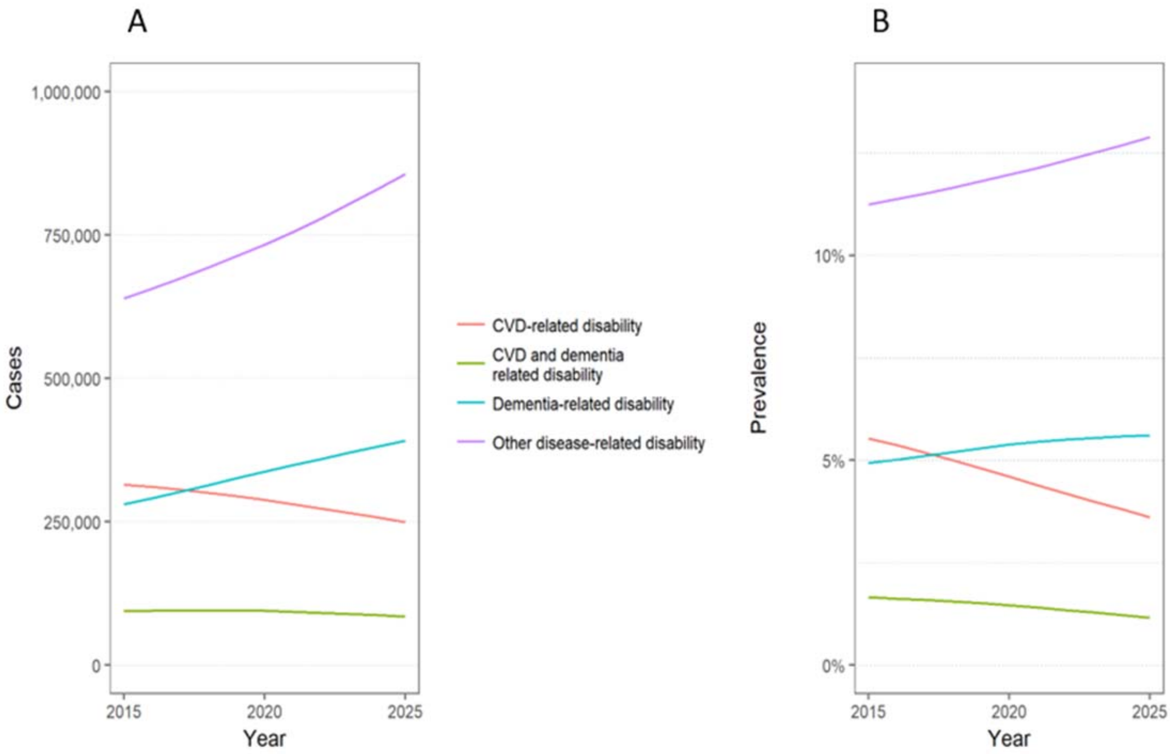

2 Sensitivity analysis

We assumed, as observed in ELSA, that the trend in CVD incidence would mirror the rate of decline of CVD mortality. We also assumed that dementia incidence would follow a 2.7% annual decline based on analysis of the incidence trends across ELSA waves (2002-2013).[1]

Due to the conflicting evidence on trends in dementia we examined two alternative assumptions on its future trend: a constant trend (no annual decline) over the time horizon (scenario 1) and, an annual decline of 4% in dementia incidence (scenario 2).

Table S.3 provides estimates of total numbers of people with disability in 2025 according to different assumptions about the annual trends in dementia incidence. Totals remain almost unchanged despite different calendar trends in incidence of dementia. However, the two alternative assumptions regarding the trend in future dementia incidence do affect the numbers in the disease-related disability states (see Figure S. 3 to Figure S. **6**). If dementia incidence remains unchanged over the next decade, the burden of dementia-related disability will increase compared to our main prediction (see dotted green lines in Figure S. **5**). This increase will be counter-balanced by a decrease in the number of cases of other types of disability, including CVD-related disability (see dotted green lines in Figure S. **4** and Figure S. **6**).

Conversely, a faster annual decline in dementia incidence of 4% would result in fewer cases of dementia-related disability (see dashed blue lines in Figure S. **5**) but an increase in the numbers of other types of disability (see dashed blue lines in Figure S. **4** and Figure S. **6**).

Table S. 4 display the estimates of healthy life expectancies at 65 in 2025 under two alternative assumptions on annual trends in dementia incidence. Notice that the proportion of life expectancy lived with disability will remain virtually unchanged from the baseline scenario for both men and woman

Table S. 3 Comparison of the numbers of disability cases (thousands) in 2025 under alternative assumptions on annual trends in dementia incidence. (95% uncertainty intervals)

	Annual trend in dementia incidence				
	2.7% annual decline (baseline)	No annual decline		4% annual decline	
	Disability cases 2025	Disability cases 2025	Difference from Baseline	Disability cases 2025	Difference from Baseline
**All**	2,811 (2,727-2,890)	2,923 (2,834-3,007)	111.5	2,803 (2,721-2,881)	-8
**Men**	1,236 (1,187-1,279)	1,283 (1,231-1,329)	47.2	1,232 (1,183-1,273)	-4.6
**Women**	1,578 (1,508-1,639)	1,641 (1,567-1,707)	63.7	1,574 (1,506-1,634)	-3.5

Figure S. 3: Predicted cases and standardised prevalence of CVD and dementia-related disability 2015-2025

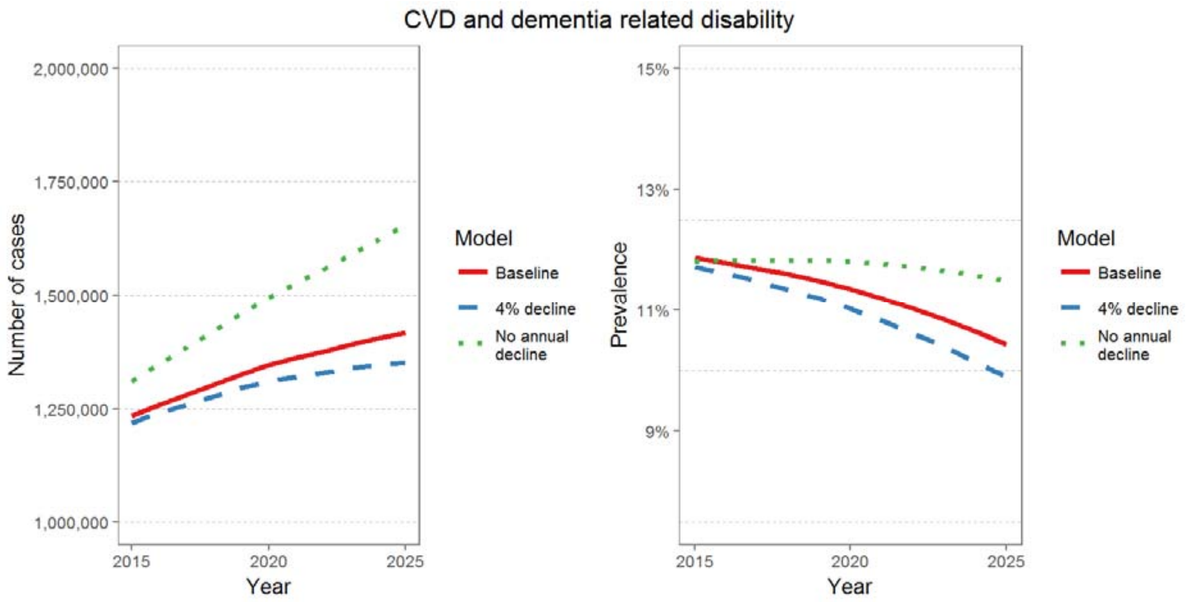

Figure S. 4: Predicted cases and standardised prevalence of CVD-related disability 2015-2025

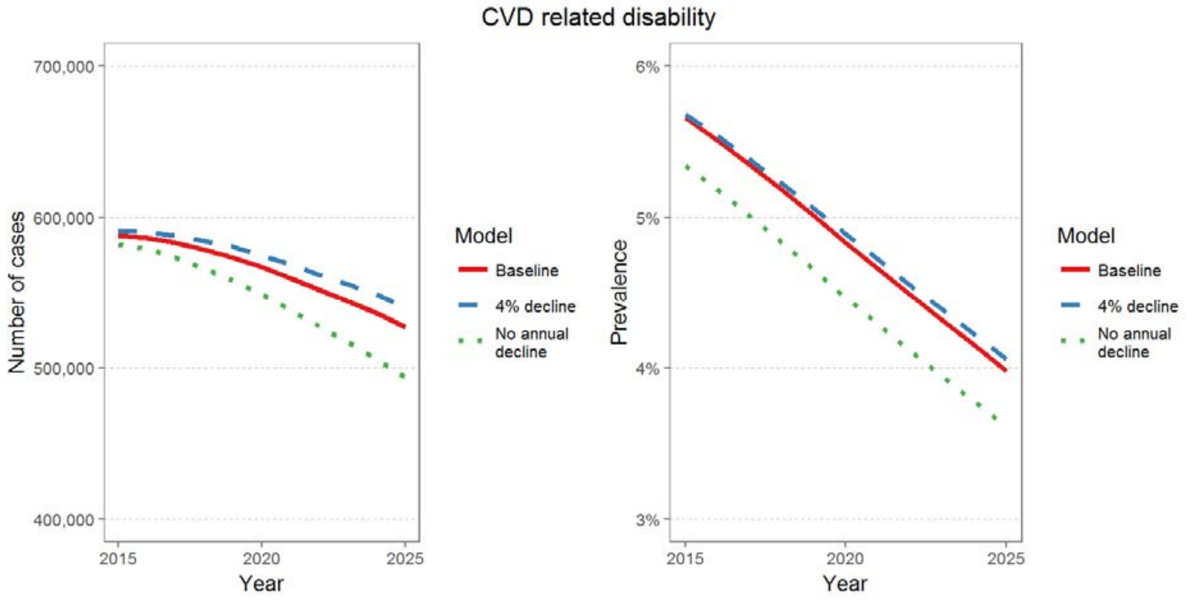

Figure S. 5: Predicted cases and standardised prevalence of dementia-related disability 2015-2025

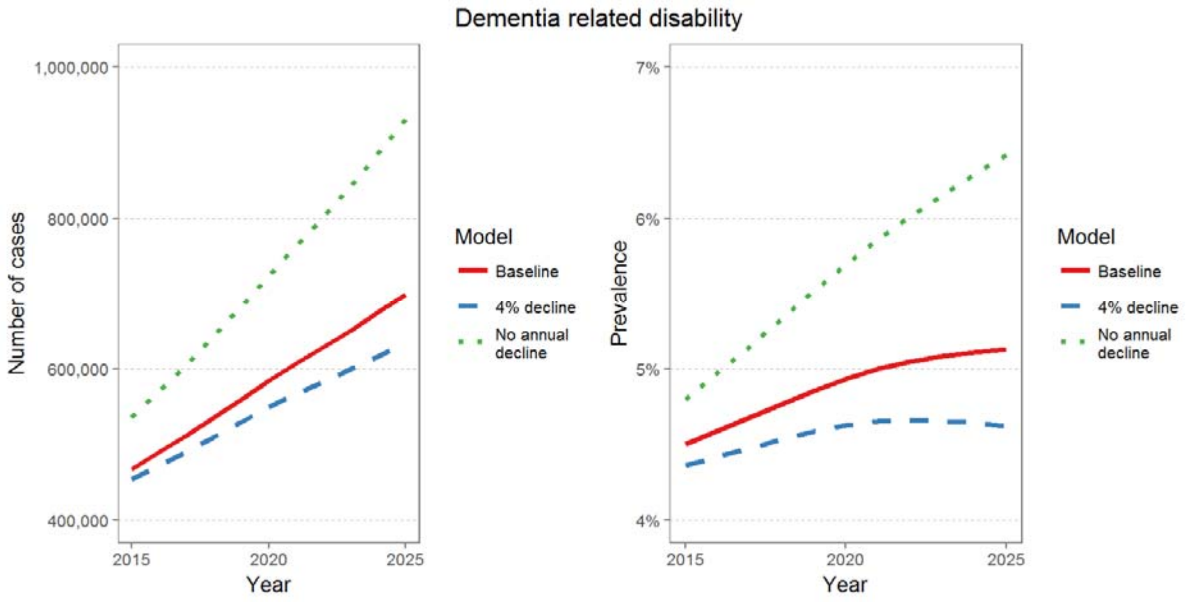

Figure S. 6: Predicted cases and standardised prevalence of Non-CVD/ Non-dementia related disability 2015-2025

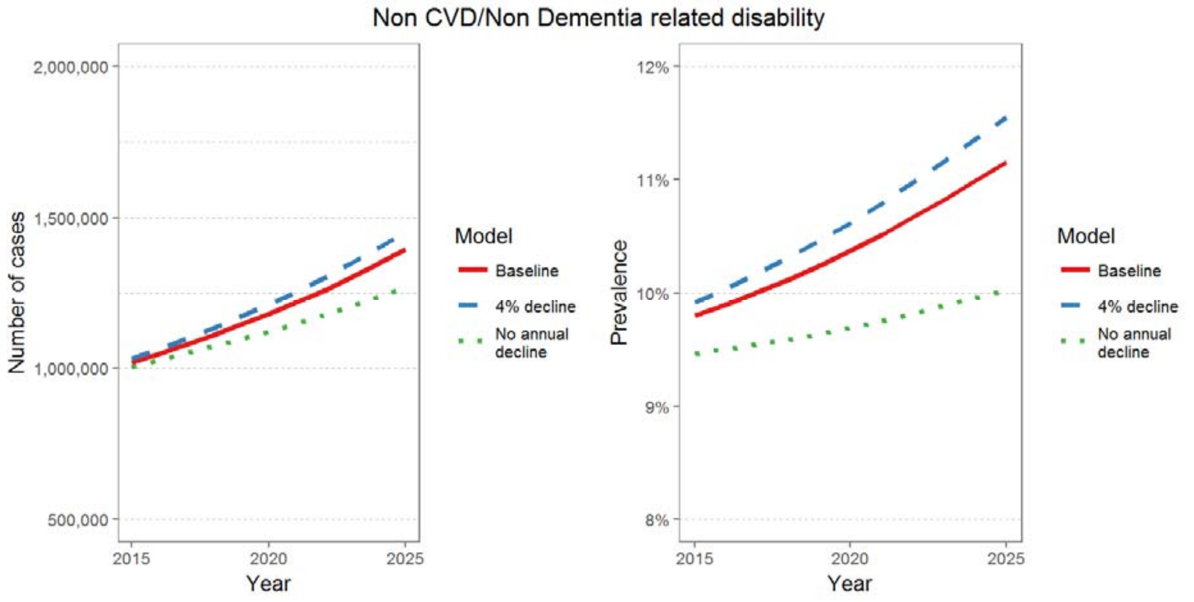

Table S. 4: Comparison of the life expectancies at 65 in 2015 under alternative assumptions on annual trends in dementia incidence (95% uncertainty intervals)

Year	2015	2025		
Annual trend in dementia incidence	Baseline (2.7% annual decline)	No annual decline	Baseline (2.7% annual decline)	4% annual decline
**Men**				
Life expectancy (LE)	19.0 (18.7-19.3)	22.4 (20.5-24.8)	21.7 (19.9-23.9)	21.9 (20.0-24.1)
Disability-free life expectancy (DFLE)	14.9 (14.7-15.1)	16.9 (15.8-18.1)	16.5 (15.4-17.6)	16.7 (15.6-17.8)
Disabled life expectancy (DLE)	4.1 (3.9-4.2)	5.5 (4.7-6.7)	5.2 (4.4-6.3)	5.2 (4.4-6.3)
Proportion (%) DLE: LE	21.4 (21.0-21.7)	24.6 (22.7-27.0)	24.0 (22.2-26.4)	23.8 (22.1-26.2)
**Women**				
Life expectancy (LE)	21.0 (20.8-21.2)	23.0 (20.4-25.7)	22.1 (19.7-24.7)	22.2 (19.8-24.8)
Disability-free life expectancy (DFLE)	15.8 (15.7-15.9)	16.9 (15.5-18.3)	16.4 (15.1-17.7)	16.5 (15.2-17.8)
Disabled life expectancy (DLE)	5.2 (5.1-5.3)	6.1 (4.9-7.6)	5.7 (4.6-7.1)	5.7 (4.7-7.1)
Proportion (%) DLE: LE	24.9 (24.5-25.2)	26.4 (24.1-29.5)	25.8 (23.5-28.9)	25.7 (23.5-28.8)

3 Research in context

We reviewed existing evidence in October 2016, searching PubMed database for any studies forecasting future trends in disability or dementia or life expectancy in the UK. The search terms used were the following:

("Dementia"[Mesh] OR "Disabled Persons"[Mesh] OR "Life Expectancy"[Mesh] OR Disab*[ti] OR Dementi*[ti] OR Longevit*[ti] OR Life expectan*[ti])

AND

("Computer Simulation"[Mesh] OR "Forecasting"[Mesh] OR "Population Forecast"[Mesh] OR Simulation*[ti] OR Model*[ti] OR forecast*[ti])

AND

("Great Britain"[MeSH Terms] OR United Kingdom[Text Word] OR "England"[ti] OR "Wales"[ti] OR "Scotland"[ti] OR "UK"[ti] OR "United Kingdom"[ti] OR "Britain"[ti])

Papers which were not relevant were manually removed. We performed additional searches using lists of references retrieved from relevant papers. The results of the search can be found in Table S. 5

Table S. 5: Results of the systematic review

Authors	Title	Population	Methods	Outcomes	Competing risks	Time Horizon
Jagger et al[2]	The effect of dementia trends and treatments on longevity and disability: a simulation model based on the MRC Cognitive Function and Ageing Study (MRC CFAS).	England	Dynamic macro-simulation	Dementia and disability prevalence	Conditional Transition probabilities (conditional on specific morbidities such as CVD)	2006-2026
Bennett et al[3]	The future of life expectancy and life expectancy inequalities in England and Wales: Bayesian spatiotemporal forecasting.	England and Wales	Bayesian spatiotemporal model	Life expectancy at a local, small area levels	No	2012-2030
Office for National Statistics [4]	Past and projected data from the period and cohort life tables: 2014-based, UK, 1981 to 2064, Office for National Statistics (2015)	United Kingdom	Mortality projections	Life expectancy	No	1981-2064

Comas-Herrera et al[5]	Cognitive impairment in older people: future demand for long-term care services and the associated costs	England	Macrosimulation	Dementia and disability	No	2002 and 2031

4 Overview of IMPACT Better Aging Model

The IMPACT Better Ageing Model (IMPACT-BAM) is a discrete-time Markov model which follows the progression of a healthy population (aged 35+ years old) from England and Wales into ten different health states characterised by the presence or absence of cardiovascular disease (CVD), cognitive impairment and functional impairment from 2006 to 2025. The model structure is presented in Figure S. 7, the health states are described in Table S. 6 and transition probabilities, $p_{i,j}$, in Table S. 7

Prior to simulation, we populated each state in the model based on ONS population estimates in 2006 (start year) and prevalence of the above conditions from ELSA, except for the new cohort of 35-year olds that enters the system through the disease-free state (see section 5.10). The simulation allows individuals to move to other states in the model. The arrows in Figure S. 7 indicate the possible movements of people between these ten states, which are governed by one-year probabilities of transition.

For example, a healthy 55-year man starts the simulation in state 1 (Disease-free state) in 2006. He moves to state 2 (CVD) in 2007 after having a stroke. In 2008 he could either die from complications of the stroke (he moves to state 9), any other causes (he moves to state 10) or he could develop cognitive impairment (moving to state 3) or disability (moving to state 5). As above, movements to any state are driven by transition probabilities. Detailed information on the estimation of transition probabilities is provided in sections 5.2 to 5.9

Then, to calculate the number of people with disability in year *t*, we count the number of people in the states that represent disability (states 5, 6, 7 and 8) at year *t*. To calculate the number of individuals with dementia, we count the number of people in state 6 and 7 at year *t*, and do so similarly for other conditions. These numbers are then used to calculate the prevalence of any disease in the model. Detailed information on output calculation can be found in section 7.

Figure S. 7: IMPACT-BAM model structure. Transitions to death states 9 and 10 are possible from any state.

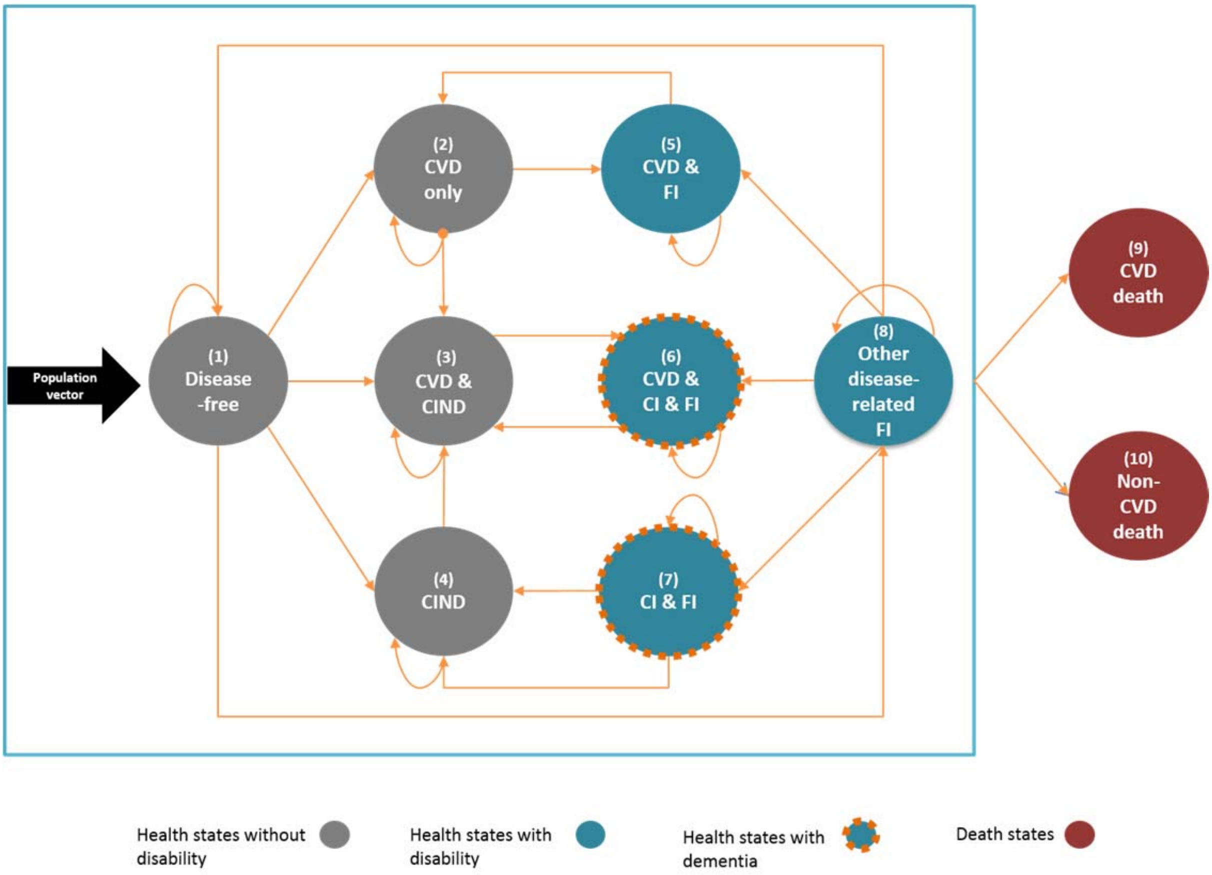

Table S. 6: Description of the health states

Health state	Name	Description
1	Disease-free population	People free of cardiovascular disease (CVD), cognitive impairment (CI) or functional impairment (FI)
2	CVD only	Cardiovascular disease
3	CVD and CIND	Cardiovascular disease and cognitive impairment no dementia
4	CIND	Cognitive impairment no dementia
5	CVD and FI	Cardiovascular disease and functional impairment
6	CVD, CIND and FI	Cardiovascular disease and dementia (cognitive + functional impairment)
7	DEMENTIA	cognitive + functional impairment
8	Other disease-related FI	Functional impairment no related to CVD or/and Dementia
9	CVD death	Death from CVD causes
10	Non-CVD death	Death from a different cause than CVD

Table S. 7: Description of transition probabilities. Each transition probability is stratified by sex and age

1-year transition probability	From	To
$p_{1,1}$	Disease-free population	Disease-free population
$p_{1,2}$	Disease-free population	CVD only
$p_{1,3}$	Disease-free population	CVD and CIND
$p_{1,4}$	Disease-free population	CIND
$p_{1,8}$	Disease-free population	Non-CVD/Non-dementia FI
$p_{1,9}$	Disease-free population	CVD death
$p_{1,10}$	Disease-free population	Non-CVD death
$p_{2,2}$	CVD only	CVD only
$p_{2,3}$	CVD only	CVD and CIND
$p_{2,5}$	CVD only	CVD and FI
$p_{2,9}$	CVD only	CVD death
$p_{2,10}$	CVD only	Non-CVD death
$p_{3,3}$	CVD and CIND	CVD and CIND
$p_{3,6}$	CVD and CIND	CVD and Dementia
$p_{3,9}$	CVD and CIND	CVD death
$p_{3,10}$	CVD and CIND	Non-CVD death
$p_{4,4}$	CIND	CIND
$p_{4,7}$	CIND	Dementia

$p_{4,9}$	CIND	CVD death
$p_{4,10}$	CIND	Non-CVD death
$p_{5,5}$	CVD and FI	CVD and FI
$p_{5,2}$	CVD and FI	CVD only
$p_{5,9}$	CVD and FI	CVD death
$p_{5,10}$	CVD and FI	Non-CVD death
$p_{6,6}$	CVD and dementia	CVD and dementia
$p_{6,3}$	CVD and dementia	CVD and CIND
$p_{6,9}$	CVD and dementia	CVD death
$p_{6,10}$	CVD and dementia	Non-CVD death
$p_{7,7}$	Dementia	Dementia
$p_{7,4}$	Dementia	CIND
$p_{7,9}$	Dementia	CVD death
$p_{7,10}$	Dementia	Non-CVD death
$p_{8,1}$	Other disease-related FI	Disease-Free population
$p_{8,5}$	Other disease-related FI	CVD and FI
$p_{8,6}$	Other disease-related FI	CVD and Dementia
$p_{8,7}$	Other disease-related FI	Dementia
$p_{8,8}$	Other disease-related FI	Non-CVD/Non-dementia FI
$p_{8,9}$	Other disease-related FI	CVD death
$p_{8,10}$	Other disease-related FI	Non-CVD death

5 Inputs and Calculations

5.1 Case definitions

Cardiovascular disease (represented in the model by states 2,3,5 and 6 in Figure S.7) was defined as having a diagnosis of cardiovascular disease, myocardial infarction, stroke and/or angina; equivalent to the ICD10 codes I00-I99, G45, Q200-Q289, M300-M319, D180-D189, A182, K550-K559, R00-R009, R071-R074, R098, R230, R590-R599 and R943.

Cognitive impairment no dementia (states 3 and 4) was defined as impairment in two or more domains of cognitive function (such as orientation to time, immediate and delayed memory, verbal fluency, and numeracy function), or a score higher than 3.6 on the Informant Questionnaire for Cognitive Decline (IQCODE)10 administered for subjects who were unable to participate in the study.[6]

Functional impairment (states, 5, 6, 7 and 8)was defined as the inability to independently perform one or more activities of daily living (ADL). The ADLs included getting in or out of bed, walking across a

room, bathing or showering, using the toilet, dressing, cutting food and eating. We distinguished four disability states: state 5 defined as CVD-related disability, state 7 as dementia-related disability, state 6 as CVD and dementia related disability and state 8 as other disease-related disability defined as other forms of disability not linked to CVD or dementia. To quantify the burden of CVD- related disability, we did not consider the contributions of state 6 (CVD and dementia related disability) as we wanted to isolate the disability burden associated to CVD only. Similarly for dementia-related disability.

Dementia (states 6 and 7) was defined based on the co-existence of cognitive impairment and functional impairment or a report of a doctor diagnosis of dementia by the participant or carer.

5.2 Incidence of CVD ($P_{1,2}$; $P_{4,3}$, $P_{8,5}$)

Denote $P(CVD)$ to be the incidence of CVD. To calculate CVD incidence, $P(CVD)$, we obtained 2-year incidence rates from the English Longitudinal Study of Ageing (ELSA) and fitted a logistic regression model of the form:

$$logit\ incidence = \beta_0 + \beta_{age} age_{35} + \beta_{sex} sex + \beta(sex * age_{35}) + \beta_{state} state$$

Where age_{35} is individual age centred at 35 and *state* are those states (states 1, 4 and 8) from where transitions to CVD states (states 2, 3 and 5) are allowed.

From the logistic regression estimates, 2-year transition probabilities were computed which were later transformed into gender specific 1-year transition probabilities for single years of age.

In our model, we defined states 2 and 4 (CVD-only and CIND-only) as mutually exclusive (i.e. a patient who is in the CVD-only state does not have CIND at the same time and vice versa). Therefore, to calculate the transition probability $p_{1,2}$ we subtract the proportion of patients who have both CVD and CIND, $p_{1,3}$

$$p_{1,2} = P(CVD) - p_{1,3}$$

5.3 Incidence of CIND ($P_{1,4}$; $P_{2,3}$, $P_{8,7}$)

Denote $P(CIND)$ to be the incidence of "cognitive impairment no dementia". To calculate CIND incidence, $P(CIND)$, 2-year incidence rates from ELSA were modelled as follows:

We fitted a logistic regression model of the form:

$$logit\ incidence = \beta_0 + \beta_{age} age_{50} + \beta_{sex} sex + \beta(sex * age_{50}) + \beta(sex * age_{50}^2) + \beta_{state} state$$

Where age_{50} is individual age centred at 50 and *state* are those states (states 1, 2 and 8) from where transitions to CI states (states 4, 3 and 7) are allowed.

This allowed us to compute 2-year transition probabilities that were later transformed into gender specific 1-year transition probabilities for single years of age. The incidence rates from ELSA are likely to be underestimated due to higher drop out of those who do develop cognitive impairment.

In our model, we defined states 2 and 4 (CVD-only and CIND-only) as mutually exclusive (i.e. a patient who is in the CVD-only state does not have CIND at the same time and vice versa). Therefore, to calculate the transition probability $p_{1,4}$, we subtract the proportion of patients who have both CVD and CIND, $p_{1,3}$

$$p_{1,4} = P(CIND) - p_{1,3}$$

5.4 Incidence of CVD and CIND ($P_{1,3}$; $P_{8,6}$)

We assume that CVD and CIND are independent events. Therefore, ($p_{1,3} = P(CVD \cap CIND)$) from a healthy state, $p_{1,3} = P(CVD) \times P(CIND)$ from above formula.

Similarly for $p_{8,6}$

5.5 Incidence of functional impairment states ($P_{1,8}$; $P_{2,5}$; $P_{3,6}$ and $P_{4,7}$)

We obtained the 2-year incidence rates for functional impairment ELSA and fitted logistic regression models of the form:

$$logit\ incidence\ FI = \beta_0 + \beta_{age} age_{35} + \beta_{sex} sex + +\beta_{state} state + \beta(sex * age_{35}) + \beta(state * age_{35})$$

Where $age_{35}$is individual age centred at 35 and *state* are those states (states 1, 2, 3 and 4) from where transitions to FI states (states 8, 5, 6 and 7) are allowed.

This allowed 2-year transition probabilities to be computed which were later transformed into gender specific1-year transition probabilities for single years of age. These transition probabilities do not have a calendar effect.

5.6 Recovery from functional impairment states ($P_{8,1}$; $P_{5,2}$; $P_{6,3}$ and $P_{7,4}$)

We obtained the 2-year incidence rates for functional impairment ELSA and fitted logistic regression models of the form:

$$logit\ incidence\ FI = \beta_0 + \beta_{age} age_{35} + \beta_{sex} sex + +\beta_{state} state + \beta(sex * age_{35}) + \beta(state * age_{35})$$

Where $age_{35}$is individual age centred at 35 and *state* are those FI states (states 8, 5, 6 and 7) from where transitions to states without FI (states 1, 2, 3 and 4) are allowed.

This allowed 2-year transition probabilities to be computed which were later transformed into gender specific1-year transition probabilities for single years of age. These transition probabilities do not have a calendar effect.

5.7 Transition probabilities from state i to the death states ($P_{i,9}$ and $P_{i,10}$)

The computation of the transition probabilities $p_{i,9}$ involved three steps:

For the first step, CVD mortality probabilities of CVD up 2025 in 5-year age bands were calculated using the Bayesian Age Period Cohort (BAPC) model,[7] with ONS mortality and population estimates from 1982-2012 for England and Wales as inputs.

The curve fitting tool in MATLAB was then used to obtain CVD mortality probabilities for single years of age, starting at 35 years old. The probabilities are estimated using piecewise cubic Hermit interpolation to estimate values that lie between known data points, with the monotonicity and the shape of the data preserved. We denote these probabilities of death by $m\_cvd_{a,t}$, where, a is the age of individual and t the calendar year.

For the second step, we calculated mortality rates from ELSA for the age groups 50-59, 60-69, 70-79 and 80-89 and fitted two logistic regression models of the form:

$$logit\ cvd\_death = \beta_0 + \beta_{age} age_{35} + \beta_{male} male + \beta(male * age_{35})$$

$$logit\ cvd\_death = \beta_0 + \beta_{age} age_{35} + \beta_{male} male + \beta(male * age_{35}) + \boldsymbol{\beta_s}\mathbf{state}$$

Where $age_{35}$is individual age centred at 35, $\boldsymbol{\beta_s}$ is a vector containing the β coefficients for all the states.

The first equation allowed us to compute gender specific baseline transition probabilities for single years of age. We defined these as $\tilde{p}_{0,9,a}$

The second equation allowed us to compute gender and state-specific transition probabilities for single years of age. We defined these as $\tilde{p}_{i,9,a}$

To estimate how different the state-specific transition probabilities are from the baseline transition probabilities we calculated $cvd_{a,i} = \frac{\tilde{p}_{i,9,a}}{\tilde{p}_{0,9,a}}$.

The probabilities of death, $m\_cvd_{a,t}$, are the probabilities of dying (from CVD) regardless of the state an individual is coming from, similar to the baseline transition probabilities $\tilde{p}_{0,9,a}$ from the ELSA study. The $m\_cvd_{a,t}$ are calculated using the entire England and Wales population and allow for cohort and calendar effects and are preferred over the $\tilde{p}_{0,9,a}$.

To allow for each subject's initial state, the $m\_cvd_{a,t}$ were multiplied by the factor $cvd_{a,i}$ to obtain the age, gender and state-specific transition probabilities $p_{i,9,a}$.

Transition probabilities $p_{i,10,a}$ were calculated in the same manner.

5.8 Calendar effect for CVD and CIND incidence

Let $\Delta_{a,t+1} = \frac{m\_cvd_{a,t+1}}{m\_cvd_{a,t}}$ where $m\_cvd_{a,t}$ is the age-specific probability of death from CVD causes in year t. Therefore, $\Delta_{a,t+1}$ is an age-specific adjustment factor describing how different the probability of CVD death in year $t+1$ is from the probability of CVD death in the previous year t.

We assume that annual changes in CVD incidence mirror the annual changes in CVD mortality as observed in ELSA (See Figure S. 8). In other words, we assume the annual percentage change in CVD incidence equals to the annual percentage change in CVD mortality. Therefore, to obtain the incidence of CVD allowing for a calendar effect, we multiplied $P(CVD)_{a,t+1}$ by $\left(\Delta_{a,t+1}\right)$.

Likewise, we assume that these annual changes in CVD incidence would also affect $p_{8,5}$, thus the same calendar was applied.

However, the incidence of CIND, $P(CIND)_{a,t+1}$, is assumed to decrease by 2.7% per calendar year, ie, $P(CIND)_{a,t+1} = 0.973 * P(CIND)_{a,t}$.

The above annual decline for CIND was estimated with data collected over 6 waves of ELSA (2002-2013) and using an elaborate model that takes into account losses to follow-up and mortality. The results of these analyses suggested that the calendar trend per year is -2.7% (95% confidence interval -2.9, -2.4).[8]

Likewise, we assume an annual 2.7% decrease for $p_{8,6}$ and $p_{8,7}$.The calculations of $p_{1,2}, p_{1,3}$ and $p_{1,4}$, proceed as previously described.

Figure S. 8: Age and sex standardised cardiovascular incidence and mortality rates in the English Longitudinal Study of Ageing 2002-2013

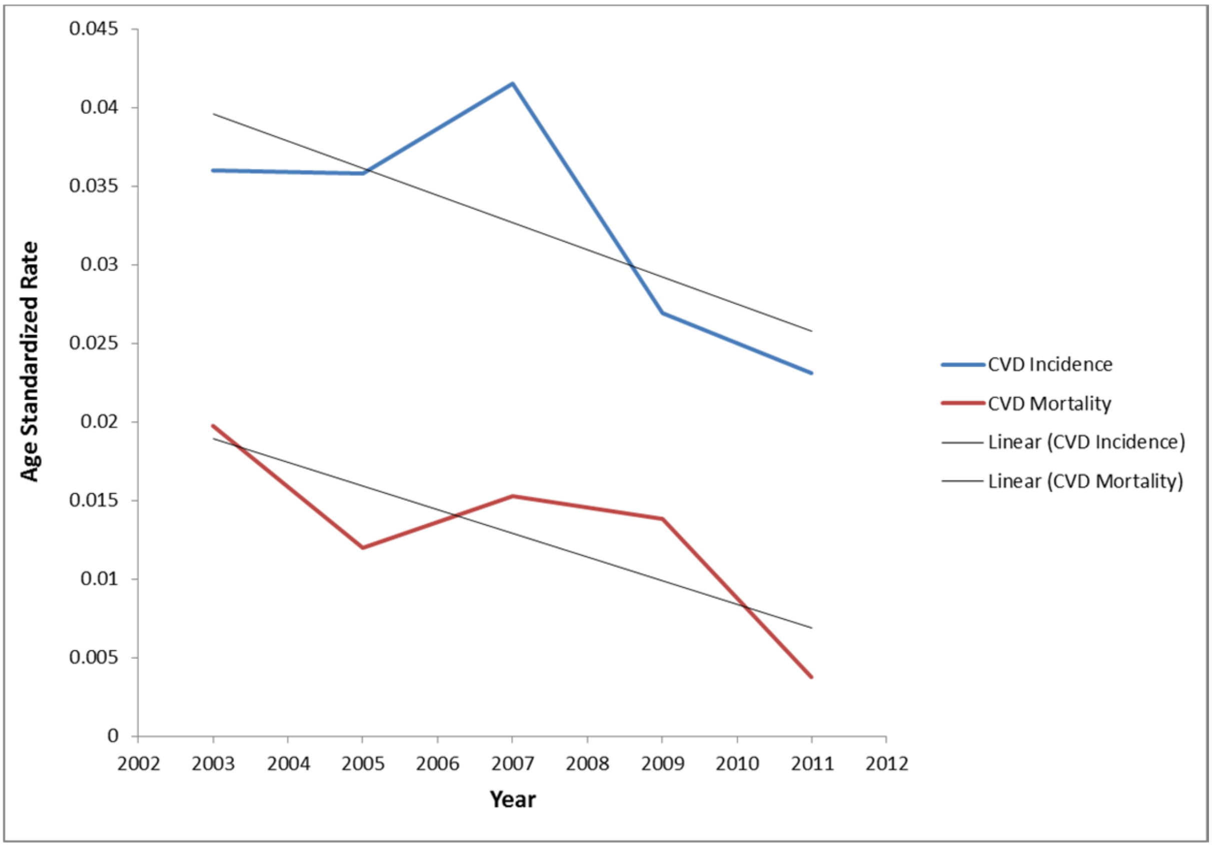

5.9 Recurrent state transition probabilities

The recurrent state transition probabilities such as $p_{1,1}$, $p_{2,2}$, $p_{3,3}$, etc. were calculated using the following formula:

$p_{i,i} = 1 - \sum_{j=1}^{J} p_{i,j}$, where J is a vector containing the states (other than i itself) to where a transition from state i is possible.

5.10 Prevalence of initial states

We obtained the 2-year prevalence rates for states 2, 3, 4, 5, 6, 7 and 8 from ELSA for 5-year age groups. Due to the small number, it was assumed that those aged <50 have a prevalence probability of cognitive impairment equal to zero. This was done by dividing the number of people in each state by the total number of individuals in that age-sex strata in the pooled ELSA data and attributed to 2006 which is the mid-point of the ELSA data collection period (2002-2013).

We then used the curve fitting tool in MATLAB to obtain data for single year of age starting at 35 years old.

ELSA contains information on 142 individuals aged 35 to 39. Approximately 97% of these individuals were free of CVD, cognitive impairment, dementia and disability. Therefore, we assumed that the new cohort of 35s entering the model at each year is free of disease. This assumption has no effect on the outputs reported in the manuscript, as the 35-year-olds entering the model in 2006 (beginning of the simulation) will be 54 years old by 2025, while the outputs reported in this study are for those 65 and older.

6 Matrix calculations

The following table contains the steps to calculate the Markov model

Table S. 8: Matrix notation for programming purposes mainly. (Example for men)

Matrix formulation (Example for men)	Description
$\mathbf{p\_m_a} = [\mathrm{p\_m}_{a,1}, \mathrm{p\_m}_{a,2}, \ldots, \mathrm{p\_m}_{a,10}]$	Column vector containing prevalence rates for all states for men aged a
$M_{a,0}$	Scalar containing initial population men aged a
$\mathrm{Tm}_{a,t} = \begin{bmatrix} p_{1,1,a,t} & \cdots & p_{1,10,a,t} \\ \vdots & \ddots & \vdots \\ p_{10,1,a,t} & \cdots & p_{10,10,a,t} \end{bmatrix}$	Matrix for men aged a, containing the transition probabilities
$\mathbf{m_{a,t}} = [\mathrm{m}_{a,t,1}, \mathrm{m}_{a,t,2}, \ldots, \mathrm{m}_{a,t,10}]$	Column vector containing the number of men aged a in each state at time t
For $t = 0$<br><br>$\mathbf{m_{a,0}} = M_{a,0} \cdot \mathbf{p\_m_a}$	
For $t = n$<br>$\mathbf{m_{a,t}} = \mathbf{m_{a-1,t-1}} \cdot [\mathrm{T}_{a-1}]^{\mathrm{T}}$<br>$\mathbf{m_{a,t}} = \begin{bmatrix} m_{a-1,t-1}p_{1,1,a-1,t-1} + m_{a-1-1,t-1}p_{2,1,a-1,t-1} + \cdots + m_{a-1,t-1}p_{10,1,a-1,t-1}, \\ m_{a-1,t-1}p_{1,2,a-1,t-1} + m_{a-1,t-1}p_{2,2,a-1,t-1} + \cdots + m_{a-1,t-1}p_{10,2,a-1,t-1}, \\ \ldots, \\ m_{a-1,t-1}p_{1,10,a-1,t-1} + m_{a-1,t-1}p_{2,10,a-1,t-1} + \cdots + m_{a-1,t-1}p_{10,10,a-1,t-1} \end{bmatrix}$	

7 Output statistics from IMPACT-BAM

The statistics generated from the IMPACT-BAM are summarised in Table S.9

Table S. 9: Description of the main output variables. (Example for men)

Output variable (Example for men)	Description	Equations
$d\_m_{a,t}$	Number of male deaths aged a at the beginning of the cycle t	$m_{a,t,9} + m_{a,t,10}$
$dis\_m_{a,t}$	Number of men with disability aged a at the beginning of the cycle t	$m_{a,t,5} + m_{a,t,6} + m_{a,t,7} + m_{a,t,8}$
$cvd\_m_{a,t}$	Number of men with CVD aged a at the beginning of the cycle t	$m_{a,t,2} + m_{a,t,3} + m_{a,t,5} + m_{a,t,6}$
$ci\_m_{a,t}$	Number of men with cognitive impairment aged a at the beginning of the cycle t	$m_{a,t,3} + m_{a,t,4} + m_{a,t,6} + m_{a,t,7}$
$deme\_m_{a,t}$	Number of male deaths aged a at the beginning of the cycle t	$m_{a,t,6} + m_{a,t,7}$
$E\_m_{a,t}$	Life expectancy for men at age a at cycle t. Using Sullivan method[5]	$s\_m_{a,t} = m_{a,t,1} + m_{a,t,2} + m_{a,t,3} + m_{a,t,4} + m_{a,t,5} + m_{a,t,6} + m_{a,t,7} + m_{a,t,8}$ $PLY\_m_{a,t} = \frac{s\_m_{a,t} + s\_m_{a+1,t}}{2}$ $Total\ PLY\_m_{a,t} = \sum_{a=a}^{A} PLY\_m_{a,t}$ $E\_m_{a,t} = \frac{Total\ PLY\_m_{a,t}}{s\_m_{a,t}}$
DLE_m_a	Disabled life expectancy for men at age a. Using Sullivan method[5]	$DPLY\_m_{a,t} = \frac{dis\_m_{a,t} + dis\_m_{a+1,t}}{2}$ $Total\ DPLY\_m_{a,t} = \sum_{a=a}^{A} DFPLY\_m_{a,t}$ $DLE\_m_{a,t} = \frac{Total\ DPLY\_m_{a,t}}{dis\_m_{a,t}}$
$DFLE\_m_a$	Disability-free life expectancy for men at age a. Using Sullivan method[9]	$dfs\_m_{a,t} = m_{a,t,1} + m_{a,t,2} + m_{a,t,3} + m_{a,t,4}$ $DFPLY\_m_{a,t} = \frac{dfs\_m_{a,t} + dfs\_m_{a+1,t}}{2}$ $Total\ DFPLY\_m_{a,t} = \sum_{a=a}^{A} DFPLY\_m_{a,t}$ $DFLE\_m_{a,t} = \frac{Total\ DFPLY\_m_{a,t}}{dfs\_m_{a,t}}$
$MFLE\_m_a$	Morbidity-free life expectancy for men at age a. Using Sullivan method[9]	$dfs\_m_{a,t} = m_{a,t,1}$ $MFLY\_m_{a,t} = \frac{m_{a,t,1} + m_{a+1,t,1}}{2}$ $Total\ MFPLY\_m_{a,t} = \sum_{a=a}^{A} MFPLY\_m_{a,t}$

		$M\dot{F}LE\_m_{a,t} = \frac{Total\ MFPLY\_m_{a,t}}{dfs\_m_{a,t}}$

8 Probability sensitivity analysis: Monte Carlo simulation

8.1 Basic Monte Carlo simulation

Let us define $\boldsymbol{\theta_{jm}}$ as a vector containing age and calendar (when applicable) specific values for the input parameter *j* at iteration *m*.

$\boldsymbol{\omega_{im}}$ as a vector containing age and calendar specific values for the output *i* at iteration *m*

$\mathbf{I_m}$ is a matrix containing all the age and calendar-specific inputs used in our Markov model at iteration *m*.

$$\mathbf{I_m} = \theta_{1m}, \theta_{2m}, \dots, \theta_{Jm}$$

$\mathbf{O_m}$ is a matrix containing all the age and calendar-specific outputs used in our Markov model at iteration *m*.

$$\mathbf{O_m} = \omega_{1m}, \omega_{2m}, \dots, \omega_{Im}$$

For 1 to M:

1. We sample θ_j from the appropriate probability distribution described in Table S. 10
2. We use the matrix $\mathbf{I_m}$ to calculate a matrix outputs $\mathbf{O_m}$ using IMPACT-BAM

   Summarise outputs in **O**: mean, median, 5th and 95th percentiles of the distribution as uncertainty intervals.

Table S. 10: Probability sensitivity analysis

Input parameters	Type of distribution and functions	Source
**Prevalence for any state**	(Beta distribution: cases, sample size minus cases)	ELSA
**Incidence of CVD**	Normal distribution (ln(mean), SE)<br>Ln(mean)=Log-scaled predicted probability from the logit model<br>SE= standard error from the predicted probability	STATA command "margins" was used after fitting the logistic model described in section 5.2 to calculate the mean predicted probability and its associated standard error
**Incidence of CIND**	Normal distribution (ln(mean), SE)<br>Ln(mean)=Log-scaled predicted probability from the logit model<br>SE= standard error from the predicted probability	STATA command "margins" was used after fitting the logistic model described in section 5.3 to calculate the mean predicted probability and its associated standard error
**Transition probabilities from/to functional impairment**	Normal distribution (ln(mean), SE)<br>Ln(mean)=Log-scaled predicted probability from the logit model<br>SE= standard error from the predicted probability	STATA command "margins" was used after fitting the logistic model described in sections 5.5 and 5.6 to calculate the mean predicted probability and its associated standard error
**Probabilities to death states**	Posterior distribution	BAMP software provided samples from the posterior distribution of the age-specific CVD and Non-CVD mortality rates

The choice of beta and normal distributions for prevalence estimates and the incidence of CVD, CIND and transition probabilities from/to functional impairment is suggested by the ISPOR-SMDM Modelling Good Research Practices Task Working Group-6.[10] The ISPOR-SMDM Modelling Good Research Practices describes recommendations for achieving transparency and validation developed by a task force appointed by the International Society for Pharmacoeconomics and Outcomes Research and the Society for Medical Decision Making.

We used a Bayesian Age-Period-Cohort model to estimate projections of probabilities of death. The Bayesian approach allows us to estimate a posterior distribution for the probabilities of death from which probabilities of death were sampled. We did not incorporate uncertainty in the ONS population projections of the cohort of 35-year olds. These principal projections are based on assumptions regarding future levels of fertility, migration and mortality which might add uncertainty to our estimates. However, population projections have proved to be relatively robust to mortality assumptions, whereas fertility and migrant variant assumptions only affect the projected numbers of children and young adults and hence, the effect on our model outputs will be very small.

9 Prevalence of disability from ELSA

Table S. 11: Percentage of the ELSA sample with disability

	All persons		Men		Women	
Age group	All	With disability (%)	All	With disability (%)	All	With disability (%)
35+	65955	10468 (15.9)	29165	4459 (15.3)	36790	6009 (16.3)
65+	32368	7206 (22.3)	14572	2976 (20.4)	17796	4230 (23.8)
35 - 39	142	4 (2.8)	30	1 (3.3)	112	3 (2.7)
40 - 44	465	17 (3.7)	107	4 (3.7)	358	13 (3.6)
45 - 49	1373	79 (5.8)	263	15 (5.7)	1110	64 (5.8)
50 - 54	7773	603 (7.8)	3150	243 (7.7)	4623	360 (7.8)
55 - 59	12321	1251 (10.2)	5633	570 (10.1)	6688	681 (10.2)
60 - 64	11513	1308 (11.4)	5410	650 (12)	6103	658 (10.8)
65 - 69	9836	1371 (13.9)	4690	649 (13.8)	5146	722 (14)
70 - 74	8510	1569 (18.4)	3986	728 (18.3)	4524	841 (18.6)
75 - 79	6540	1527 (23.3)	2941	647 (22)	3599	880 (24.5)
80 - 84	4315	1327 (30.8)	1794	486 (27.1)	2521	841 (33.4)
85 - 89	2277	904 (39.7)	876	317 (36.2)	1401	587 (41.9)
90+	890	508 (57.1)	285	149 (52.3)	605	359 (59.3)

Table S. 12: Percentage of cases of disability in ELSA with additional CVD and/or cognitive impairment

	All persons		Men		Women	
Age group	Disability Cases	With CVD and/or CI (%)	Disability Cases	With CVD and/or CI (%)	Disability Cases	With CVD and/or CI (%)
35+	10468	4712 (45)	4459	2154 (48.3)	6009	2558 (42.6)
65+	7206	3831 (53.2)	2976	1637 (55)	4230	2194 (51.9)
35 - 39	4	0 (0)	1	0 (0)	3	0 (0)
40 - 44	17	0 (0)	4	0 (0)	13	0 (0)
45 - 49	79	11 (13.9)	15	4 (26.7)	64	7 (10.9)
50 - 54	603	141 (23.4)	243	71 (29.2)	360	70 (19.4)

**55 - 59**	1251	343 (27.4)	570	209 (36.7)	681	134 (19.7)
**60 - 64**	1308	386 (29.5)	650	233 (35.8)	658	153 (23.3)
**65 - 69**	1371	528 (38.5)	649	265 (40.8)	722	263 (36.4)
**70 - 74**	1569	699 (44.6)	728	354 (48.6)	841	345 (41)
**75 - 79**	1527	796 (52.1)	647	369 (57)	880	427 (48.5)
**80 - 84**	1327	812 (61.2)	486	320 (65.8)	841	492 (58.5)
**85 - 89**	904	624 (69)	317	217 (68.5)	587	407 (69.3)
**90+**	508	372 (73.2)	149	112 (75.2)	359	260 (72.4)

10 Validation of the model

We validated key model outputs against empirical observations using a graphical approach and by checking whether our estimates fall within the reported 95% confidence intervals (when available).

We carried out partially-dependent validation of our estimates of CVD and Non-CVD deaths with observed ONS mortality data reported for the period 2006-2012. It is defined partially-dependent validation as this source was used to build a part of the model, but it does not wholly determine the outcome to be validated.[11] The model provided a good match to the ONS estimates of the number of CVD and Non-CVD deaths (Figure S. 9 and Figure S. 10).

We carried out independent validation (i.e. no information from these sources was used to build the model) of our model estimates of prevalence of CVD, disability and dementia, and life expectancy at age 65. Our estimates of CVD in 2011 for men fall within the 95% confidence intervals reported by the HSE (Figure S. 11). However, our model estimates higher prevalence of CVD in women. Our estimates of disability prevalence in 2014 for women fall within the 95% confidence intervals reported by ELSA wave 7 (Figure S. 12), but our model estimates a slighthly lower prevalence of disability in men.

Our age-specific estimates of dementia prevalence in 2011 were akin to those reported in CFAS II for the same year (Figure S. 13). All our estimates of our age-specific prevalence estimates fall within the 95% confidence interval reported by CFAS II, except men 80-84 and women 85-89, which were both outside by a very narrow margin.

Our estimates of LE at 65 for the period 2006-2012 were close to those reported by the ONS and EHLEIS (see Table S. 13). We also compare our projections of life expectancy at 65 with ONS projections and two studies published in the Lancet (only years 2025 and 2030 were available for comparison).[3,12]

All sources of comparison reported increases in life expectancy at 65 and all previous estimates lie within IMPACT-BAM's 95% credible intervals (Table S.14). The small differences between these studies and our estimates may be explained by the methodology used to calculate mortality

projections. The ONS makes assumptions on future improvements of mortality based on expert judgment.[13] Bennet et al. and Kontis et al. use demographic models that incorporate data on age and birth cohort in mortality trends. Our model uses further information on disease specific mortality projections,[14] specifically the combined effects of cardiovascular disease, dementia, disability and mortality to predict life expectancy. Our conservative estimates for women compared to other studies may be explained by the fact that our model predicts that more women than men will die due to causes related to their higher prevalence of dementia and functional limitations. Among men, our estimates of life expectancy in men are sensitive to the declining trends in CVD incidence and mortality observed in ELSA over the past decade and projected forward in our model.

In this section, comparisons should be made cautiously as the methodologies and underlying assumptions from the other sources are not directly comparable with ours.

10.1 CVD mortality

Figure S. 9: Predicted CVD mortality against ONS estimates 2006-2012

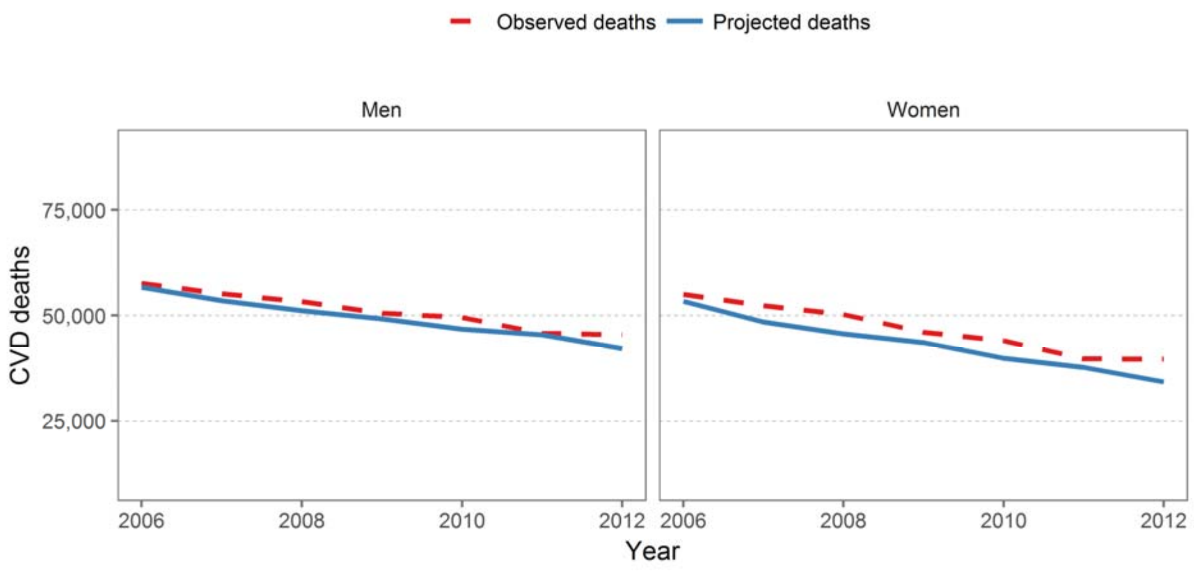

10.2 Non-CVD mortality

Figure S. 10: Predicted Non-CVD mortality against ONS estimates 2006-2012

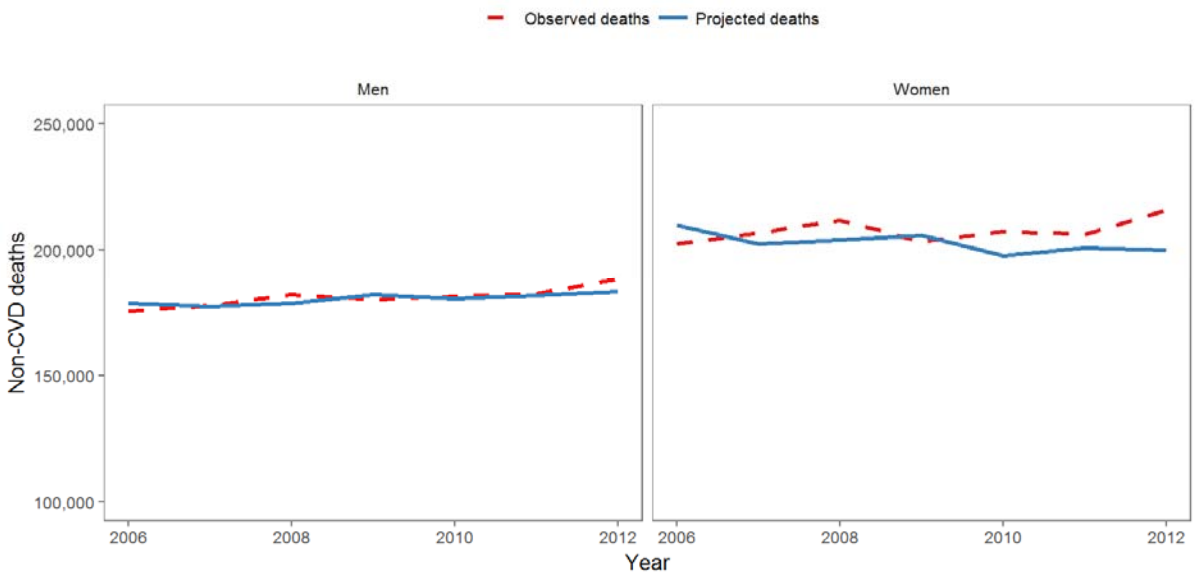

10.3 Prevalence of CVD

Figure S. 11: Predicted prevalence of CVD against Health Survey for England estimates in 2011. The error bars represent 95% uncertainty intervals for IMPACT-BAM predictions and 95% confidence intervals for HSE estimates.

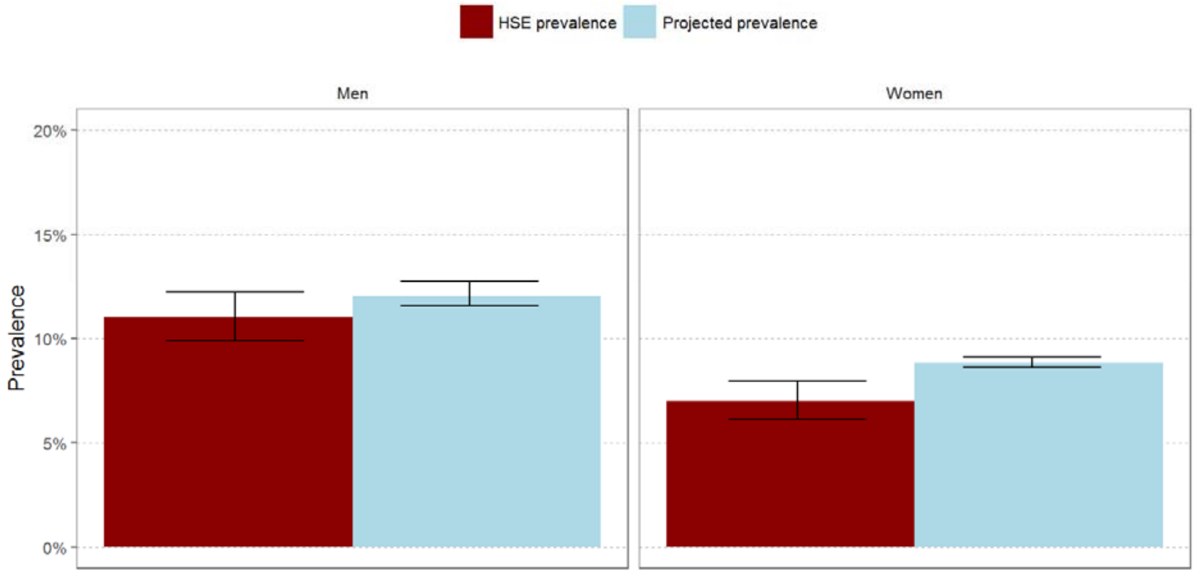

10.4 Prevalence of disability

Figure S. 12: Predicted prevalence of disability against wave 7 of ELSA. The error bars represent 95% uncertainty intervals for IMPACT-BAM predictions and 95% confidence intervals for ELSA estimates

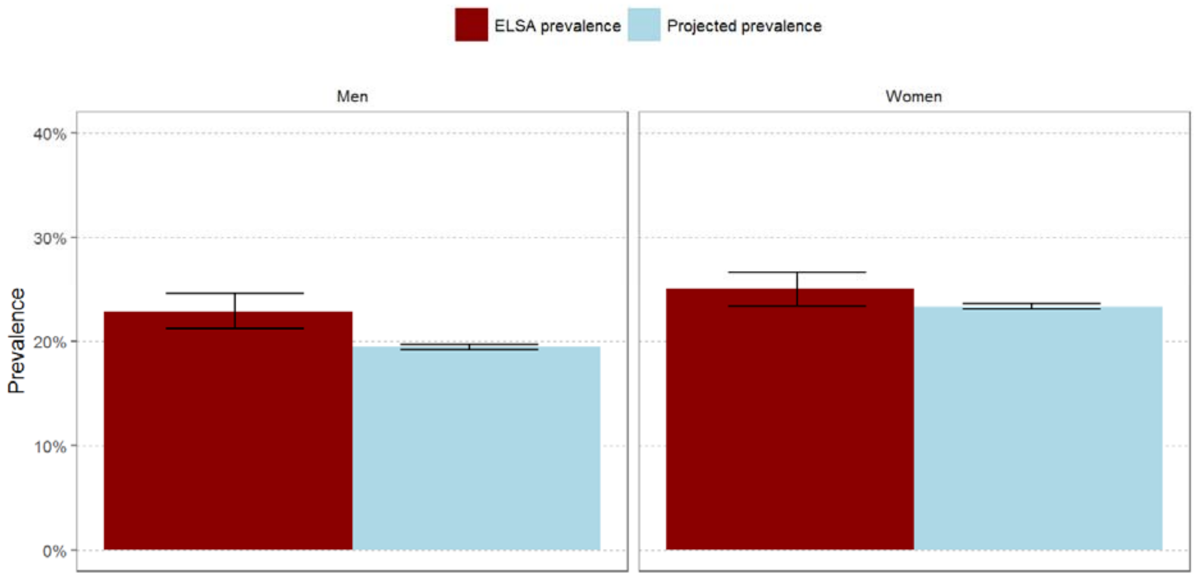

10.5 Prevalence of dementia

Figure S. 13: Age and gender specific predicted prevalence of dementia against CFAS estimates in 2011[1]. The error bars represent 95% uncertainty intervals for IMPACT-BAM predictions and 95% confidence intervals for CFAS II estimates.

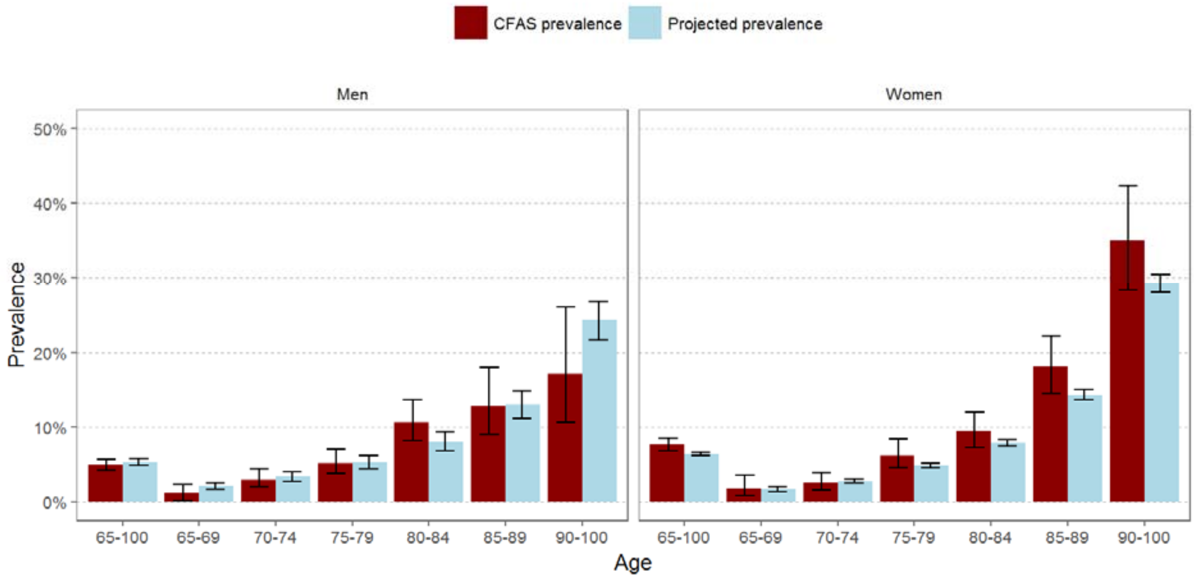

10.6 Life expectancy

Table S. 13: Comparison of LE at 65 in our model against ONS and EHLEIS estimates 2006-2012

		2006	2007	2008	2009	2010	2011	2012
**Men**	**IMPACT-BAM for England and**	16.6	16.9	17.1	17.2	17.6	17.8	18.1

	**Wales**							
	**ONS estimates for England and Wales**	17.3	17.5	17.6	17.9	18.1	18.4	18.4
	**EHLEIS for UK[1]**	17.3	17.5	17.6	18.0	18.2	18.5	18.5
**Women**	**IMPACT-BAM for England and Wales**	19.2	19.6	19.8	19.9	20.4	20.5	20.8
	**ONS estimates for England and Wales**	20.1	20.2	20.2	20.7	20.7	21.0	20.9
	**EHLEIS for UK[1]**	20.1	20.2	20.2	20.7	20.8	21.1	20.9

[1] EHLEIS only reports figures for the United Kingdom. However, their estimates are also very close to those reported by the ONS for England and Wales

Table S. 14: Comparison of LE at 65 in our model against published projected for 2025 and 2030. ** 95% credible intervals in parenthesis. [++] Although no reported in this study, our model can produce estimates for 2030. [#]these estimates are an approximation since the data was presented in graphical format only.

	**Men**		**Women**	
	**2025**	**2030**	**2025**	**2030**
**IMPACT-BAM for England and Wales[++]**	21.7 (19.9-23.9)	22.8 (19.6-27.3)	22.1 (19.7-24.7)	22.3 (18.8-27.6)
**ONS for England and Wales**	21.1	21.7	23.4	24.1
**Bennet et al (2015) for England and Wales[#]**	22.0	23.2	24.1	24.9
**Kontis et al (2017) for UK**	NA	20.9 (17.2-23.0)	NA	22.7 (18.8-24.7)

11 References

1 Steptoe A, Breeze E, Banks J, Nazroo J. Cohort profile: the English longitudinal study of ageing. *Int J Epidemiol* 2013; **42**: 1640–8.

2 Jagger C, Matthews R, Lindesay J, Robinson T, Croft P, Brayne C. The effect of dementia trends and treatments on longevity and disability: a simulation model based on the MRC Cognitive Function and Ageing Study (MRC CFAS). *Age Ageing* 2009; **38**: 319–25.

3 Bennett JE, Li G, Foreman K, *et al.* The future of life expectancy and life expectancy inequalities in England and Wales: Bayesian spatiotemporal forecasting. *Lancet* 2015; **386**: 163–70.

4 Office for National Statistics. Past and projected data from the period and cohort life tables, 2014-based, UK, 1981 to 2064. 2015. www.ons.gov.uk/peoplepopulationandcommunity/birthsdeathsandmarriages/lifeexpectancies/bulletins/pastandprojecteddatafromtheperiodandcohortlifetables/2014baseduk1981to2064/pdf (accessed Jan 15, 2016).

5 Comas-Herrera A, Wittenberg R, Pickard L, Knapp M. Cognitive impairment in older people: future demand for long-term care services and the associated costs. *Int J Geriatr Psychiatry* 2007; **22**: 1037–45.

6 Harrison JK, Fearon P, Noel-Storr AH, McShane R, Stott DJ, Quinn TJ. Informant Questionnaire on Cognitive Decline in the Elderly (IQCODE) for the diagnosis of dementia within a secondary care setting. Cochrane Database Syst. Rev. 2015.

7 Guzman-Castillo M, Gillespie DOS, Allen K, *et al.* Future declines of coronary heart disease mortality in england and wales could counter the burden of population ageing. *PLoS One* 2014; **9**: e99482.

8 Ahmadi-Abhari S, Guzman Castillo M, Bandosz P, *et al.* OP25 Dementia prevalence projections to 2030 for England and Wales: IMPACT-Better Ageing Model. *J Epidemiol Community Health* 2016; **70**: A18–A18.

9 Sullivan DF. A single index of mortality and morbidity. *HSMHA Health Rep* 1971; **86**: 347–54.

10 Briggs AH, Weinstein MC, Fenwick EAL, Karnon J, Sculpher MJ, Paltiel AD. Model Parameter Estimation and Uncertainty Analysis: A Report of the ISPOR-SMDM Modeling Good Research Practices Task Force Working Group–6. *Med Decis Mak* 2012; **32**: 722–32.

11 Eddy DM, Hollingworth W, Caro JJ, Tsevat J, McDonald KM, Wong JB. Model transparency and validation: a report of the ISPOR-SMDM Modeling Good Research Practices Task Force–7. *Med Decis Mak* 2012; **32**: 733–43.

12 Kontis V, Bennett JE, Mathers CD, Li G, Foreman K, Ezzati M. Future life expectancy in 35 industrialised countries: projections with a Bayesian model ensemble. *Lancet* 2017; published online March 24. DOI:10.1016/S0140-6736(16)32381-9.

13 ONS. Period and cohort life expectancy explained: Guide to the two types of life table. 2016

https://www.ons.gov.uk/peoplepopulationandcommunity/birthsdeathsandmarriages/lifeexpectancies/methodologies/periodandcohortlifeexpectancyexplained.

14 Booth H, Tickle L. Mortality modelling and forecasting: A review of methods. *Ann Actuar Sci* 2008; **3**: 3–43.
